# Identification and quantitation of the actual active components in bamboo juice and its oral liquid by NMR and UPLC-Q-TOF-MS

**DOI:** 10.1038/s41598-020-76897-3

**Published:** 2020-11-12

**Authors:** Quan Gao, Detao Wang, Siyue Shao, Yingying Xue, Yingfang Zhang, Chen Chen, Feng Tang, Jia Sun, Yansu Li, Qirong Guo

**Affiliations:** 1 School of Plant Protection, Anhui Agricultural University, Hefei, 230036 China; 2 State Forestry Administration Key Open Laboratory, International Centre for Bamboo and Rattan, Beijing, 100102 China; 3 Institute of Vegetables and Flowers, The Chinese Academy of Agricultural Sciences, Beijing, 100081 China; 4 College of Forestry, Nanjing Forestry University, Nanjing, 210037 China

**Keywords:** Analytical chemistry, Photochemistry

## Abstract

Bamboo juice is a traditional Chinese drink and herbal medicine, and bamboo juice oral liquids are widely sold for the treatment of cough and phlegm in China. In this study, 26 main compounds of bamboo juice (*Phyllostachys edulis*) were separated, precisely identified, and qualitative analysis using NMR (nuclear magnetic resonance) and quantitative analysis using UPLC-Q-TOF-MS (ultra-performance liquid chromatography with high-resolution quadrupole time-of-flight mass spectrometer), respectively. Potentially harmful levels of added excessive preservatives, including benzoic acid, ethylparaben, and sorbic acid, were found in bamboo juice oral liquid. Carbohydrates were determined to be the major components of bamboo juice, with contents as high as 191.13 g L^−1^, far higher than those of other compounds. The result indicated that the cough relief activity of bamboo juice oral liquid may be related to their high levels of added preservatives.

## Introduction

Bamboo juice is the sap from fresh poles of Gramineae plants such as pink green bamboo (*Phyllostachys glauca*), moso bamboo (*Phyllostachys edulis*), and other bamboo species^1^. In Chinese culture, bamboo juice, which is known as “zhuli,” is consumed as an herbal medicine and natural beverage. Additionally, bamboo juice oral liquids (BJOL) are well known for as treatments for cough and phlegm in China, and can be bought in almost any pharmacy^2^. Numerous bamboo juice oral liquids are sold as herbal medicines in the form of a decoction in the Chinese market^3–5^.

At present, few studies dealing with the components of bamboo juice have been published. Known bamboo juice components determined using gas chromatography/mass spectrometry (GC/MS) include phenols, amino acids, inorganic elements, and organic acids^6^. In some reports, the main ingredients of bamboo juice were reported to be amino acids and guaiacol^7,8^. Although the specific bamboo juice components responsible for treating cough and phlegm have not been convincingly identified, bamboo juice oral liquids sold on the Chinese market are considered effective for cough relief^9^. In Chinese hospitals, doctors often prescribe BJOL as a natural traditional herbal medicine to patients who have cough and asthma, especially to children and pregnant women.

Therefore, this study focused on the qualitative and quantitative analysis of the main components of fresh bamboo juice and bamboo juice oral liquid, with the aim to discover the activity compounds from bamboo juice to treat coughs. We have analysed nine of the most common BJOL products that are classified as “fresh bamboo juice oral liquid” with the National Medical Products Administration of China. Surprisingly, bamboo juice oral liquids on the Chinese market were found to contain levels of preservatives that might be potentially harmful to the human body. Considering the important role of BJOLs in daily Chinese life, these potentially harmful levels of preservatives represent a potential food safety problem. Additionally, the carbohydrate species and content of bamboo juice and BJOLs were analysed, and the potential function and utilization of bamboo juice is discussed.

## Results and discussion

### Isolation and identification of the main compounds from fresh bamboo juice

**Figure 1:**
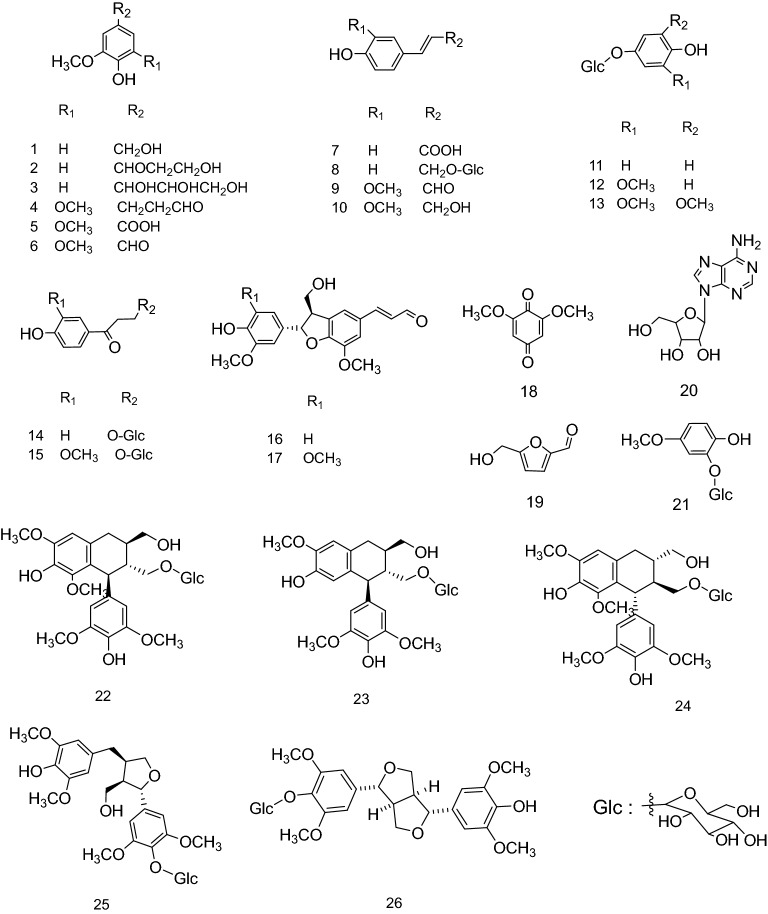
Compound **1**–**26** isolated from fresh bamboo juice.

Twenty-six compounds were obtained by column chromatography (Fig. 1). The compounds were identified as follows by comparison of their spectroscopic and physical data with those previously reported in the literature: 3-methoxy-4-hydroxybenzyl alcohol (**1**)^10^, *β*-hydroxypropiovanillone (**2**)^11^, 1-(4-hydroxy-3-methoxyphenyl)-glycerol (**3**)^12^, 3,5-dimethoxy-4-hydroxydihydrocinnamaldehyde (**4**)^13^, syringate (**5**)^14^, syringic aldehyde (**6**)^15^, *p*-*E*-coumaric acid (**7**)^16^, triandrin (**8**)^17^, ferulaldehyde (**9**)^18^, coniferol (**10**)^19^, *β*-arbutin (**11**)^20^, tachioside (**12**)^21^, 4-hydroxy-3,5-dimethoxyphenyl-*β*-d-glucopyranoside (**13**)^22^, 3,4′-dihydroxy-propiophenone-3-*β*-d-glucopyranoside (**14**)^23^, *β*-hydroxypropiovanillone (**15**)^24^, (+)-balanophonin (**16**)^25^, (+)-5-methoxybalanophonin (**17**)^25^, 2,6-dimethoxy-*p*-benzoquinone (**18**)^26^, 5-(hydroxymethyl)-2-furaldehyde (**19**)^27^, adenosine (**20**)^28^, 2-hydroxy-5-methoxyphenyl-*β*-d-glucopyranoside (**21**)^29^, (+)-lyoniresinol-3*α*-*O*-*β*-d-glucopyranosyl (**22**)^30^, (+)-5′-methoxyisolariciresinol-3*α*-*O*-*β*-d-glucopyranoside (**23**)^30^, (−)-lyoniresinol-3*α*-*O*-*β*-d-glucopyranoside (**24**)^30^, 7*S*,8*R*,8′*R*-5, 5′-dimethoxyariciresinol-4-*O*-*β*-d-glucopyranoside (**25**)^31^, and syringaresinol-4′-*O*-*β*-glucopyranoside (**26**)^32^. Purified UV images, NMR, and MS data for all compounds are shown in the Supplementary information.

#### Methodological verification

The LOD, LOQ, accuracy, and intraday and inter-day precision data are presented in Table S1 (Supplementary information). The detection and quantitation limits of the 26 components were within the appropriate range. The relative standard deviations of the accuracy and intraday and inter-day precision studies were less than 3%, indicating that the results of sample analyses performed within 4 days are reliable^33^. The recoveries of different compounds were also tested and are presented in Table S2 (Supplementary information). According to the results, the average recovery of the 26 components was more than 85%. The results show that the method can be used for the quantitative determination of the main components of bamboo juice.

#### Quantitative analysis of the main components of fresh bamboo juice

**Figure 2:**
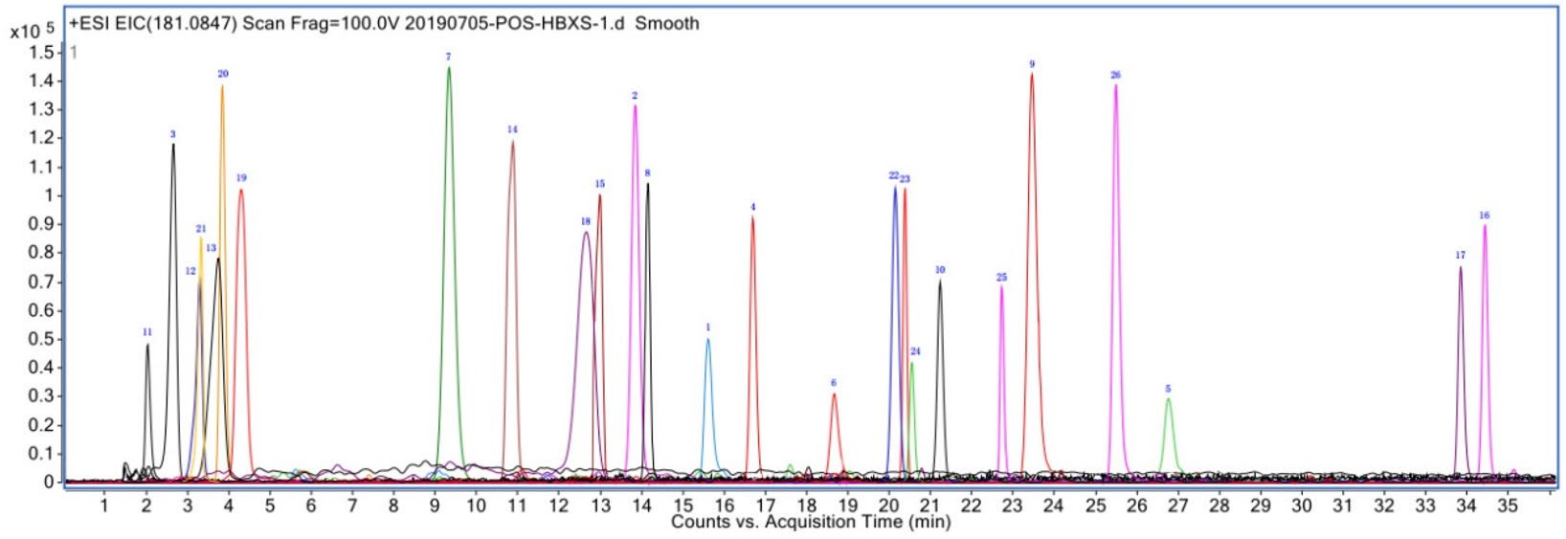
UPLC-Q-TOF-MS chromatogram of the mixed reference containing the 26 components.

**Figure 3:**
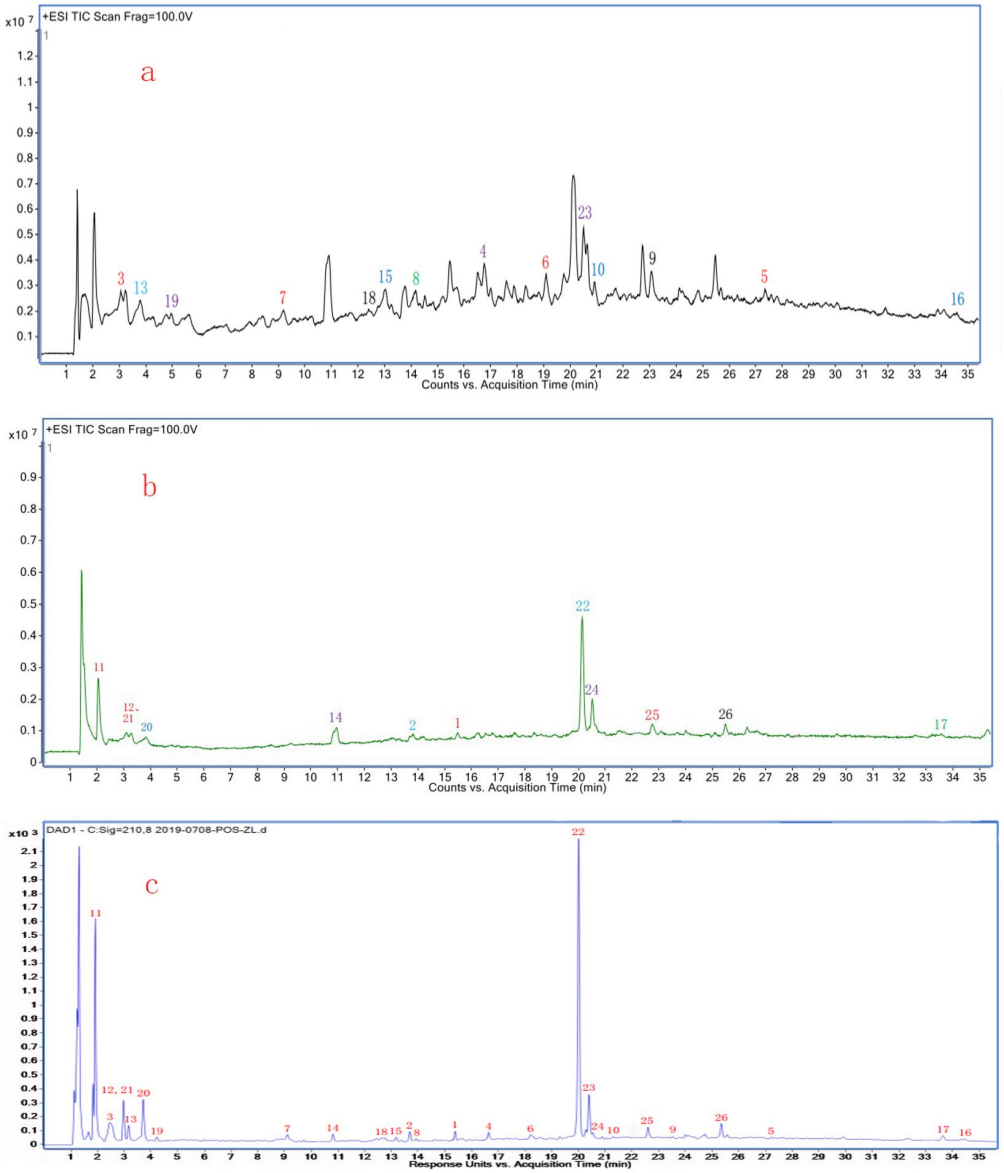
UPLC-Q-TOF-MS and DAD chromatogram of fresh bamboo juice. (**a**) The TIC diagram of fresh bamboo juice diluted 10 times. (**b**) The TIC diagram of fresh bamboo juice diluted 100 times. (**c**) The DAD detection diagram of fresh bamboo juice.

A typical chromatogram obtained from the mixed standards is presented in Fig. 2. The linear relationships observed are presented in Table S3 (Supplementary information). The standard curves exhibited good linearity over the corresponding ranges. The chromatogram of fresh bamboo juice extract is shown in Fig. 3. After validating the method, fresh bamboo juice was quantitatively analysed. The contents of all compounds are summarised in Table 1. In fresh bamboo juice diluted 10 times, the content of compound **13** was 20.71 ± 0.11 mg L^−1^. In fresh bamboo juice diluted 100 times, the content of compound **12** was 35.33 ± 0.10 mg L^−1^, and that of compound **22** was 47.15 ± 0.06 mg L^−1^.

**Table 1:** Results of content of 26 compounds in fresh bamboo juice and BJOLs.

Compound	Fresh bamboo juice (mg L^−1^)^a^	BJOL-1 (mg L^−1^)^a^	BJOL-2 (mg L^−1^)^a^	BJOL-3 (mg L^−1^)^a^	BJOL-4 (mg L^−1^)^a^	BJOL-5 (mg L^−1^)^a^	BJOL-6 (mg L^−1^)^a^	BJOL-7 (mg L^−1^)^a^	BJOL-8 (mg L^−1^)^a^	BJOL-9 (mg L^−1^)^a^
**1**	2.28 ± 0.037	0	0	0	0	0	0	0	0	0
**2**	0.41 ± 0.025	0.008 ± 0.03	0.12 ± 0.02	0.10 ± 0.01	0.35 ± 0.02	0.37 ± 0.02	0	0	0	0.051 ± 0.001
**3**	0.14 ± 0.031	0.92 ± 0.025	0.55 ± 0.02	0.46 ± 0.02	0.39 ± 0.12	0.55 ± 0.06	0.028 ± 0.01	0.027 ± 0.001	0.014 ± 0.001	0.027 ± 0.003
**4**	2.13 ± 0.015	0	0	0	0	0	0	0	0	0
**5**	4.07 ± 0.067	0	0	0	0	0	0	0	0	0
**6**	1.41 ± 0.067	0	0	0	1.38 ± 0.03	0	0	0	0	0
**7**	7.26 ± 0.12	0	0	0	0	0	0	0	0	0
**8**	1.66 ± 0.062	0	0	0	0.62 ± 0.01	0.96 ± 0.05	0	0	0	0
**9**	7.21 ± 0.015	0.25 ± 0.02	1.89 ± 0.047	0.61 ± 0.02	0.73 ± 0.03	1.22 ± 0.02	0	0	0	0
**10**	5.01 ± 0.021	0	0	0	0	0	0	0	0	0
**11**	15.15 ± 0.12	0.039 ± 0.005	0.068 ± 0.002	0.096 ± 0.001	0.51 ± 0.02	0.39 ± 0.03	0.051 ± 0.01	0	0.14 ± 0.011	0.02 ± 0.001
**12**	35.33 ± 0.10	2.81 ± 0.065	0.23 ± 0.015	2.97 ± 0.04	8.14 ± 0.02	29.04 ± 0.11	0	2.36 ± 0.031	0	1.37 ± 0.101
**13**	20.71 ± 0.11	2.38 ± 0.060	3.86 ± 0.032	5.21 ± 0.03	10.23 ± 0.01	14.51 ± 0.18	0	4.61 ± 0.015	0	12.44 ± 0.065
**14**	12.68 ± 0.13	0.25 ± 0.026	0	0	0.15 ± 0.02	1.32 ± 0.04	0	0.048 ± 0.001	0	0.032 ± 0.003
**15**	7.34 ± 0.073	0	0	0	0	0.59 ± 0.03	0	0	0	0
**16**	1.06 ± 0.051	0.031 ± 0.001	0.18 ± 0.025	0.16 ± 0.01	0.13 ± 0.02	0.51 ± 0.01	0	0	0	0
**17**	1.69 ± 0.043	0.091 ± 0.002	0.41 ± 0.022	0.45 ± 0.02	0.43 ± 0.02	1.09 ± 0.04	0	0	0	0
**18**	1.71 ± 0.025	0.076 ± 0.002	0.14 ± 0.015	0.078 ± 0.002	1.07 ± 0.03	2.15 ± 0.13	0	0.19 ± 0.025	0	0.026 ± 0.002
**19**	2.92 ± 0.041	0.88 ± 0.043	4.64 ± 0.031	17.54 ± 0.02	6.81 ± 0.02	16.48 ± 0.22	0	0.12 ± 0.011	0	0
**20**	0.62 ± 0.025	0.35 ± 0.015	0.49 ± 0.041	0.61 ± 0.02	0.97 ± 0.03	1.41 ± 0.02	0	4.01 ± 0.031	0	8.53 ± 0.04
**21**	16.61 ± 0.07	0.96 ± 0.71	2.43 ± 0.015	3.12 ± 0.02	5.16 ± 0.04	5.78 ± 0.07	0	2.14 ± 0.021	0	5.32 ± 0.11
**22**	47.15 ± 0.06	2.65 ± 0.08	4.91 ± 0.052	5.19 ± 0.03	20.87 ± 0.11	34.49 ± 0.83	0	0	0	0
**23**	17.67 ± 0.11	0	0	0	0	2.91 ± 0.09	0	0.026 ± 0.003	0	2.37 ± 0.11
**24**	14.47 ± 0.15	2.30 ± 0.025	4.04 ± 0.065	4.09 ± 0.03	2.98 ± 0.03	5.03 ± 0.16	6.52 ± 0.03	5.26 ± 0.041	5.02 ± 0.036	3.33 ± 0.055
**25**	3.69 ± 0.043	0.64 ± 0.015	0.93 ± 0.049	0.59 ± 0.03	0.31 ± 0.02	1.05 ± 0.03	1.91 ± 0.02	2.06 ± 0.052	1.79 ± 0.046	1.40 ± 0.11
**26**	6.74 ± 0.049	0.0326 ± 0.04	0.089 ± 0.007	0.034 ± 0.001	4.31 ± 0.01	8.07 ± 0.02	0	0.41 ± 0.014	0	0.68 ± 0.01

^a^Mean ± SD, *n* = 3.

A standard full dose of bamboo juice for an adult is about 60 mL per day; the corresponding average doses of each of the main compounds would thus be about 2.8 mg or less than 3 mg, which could be speculated that the dose is low to provide cough relief activity. Moreover, these compounds do not appear to show good anti-inflammatory activity.

#### Quantitative analysis of the main compounds and preservatives in bamboo juice oral liquids

**Figure 4:**
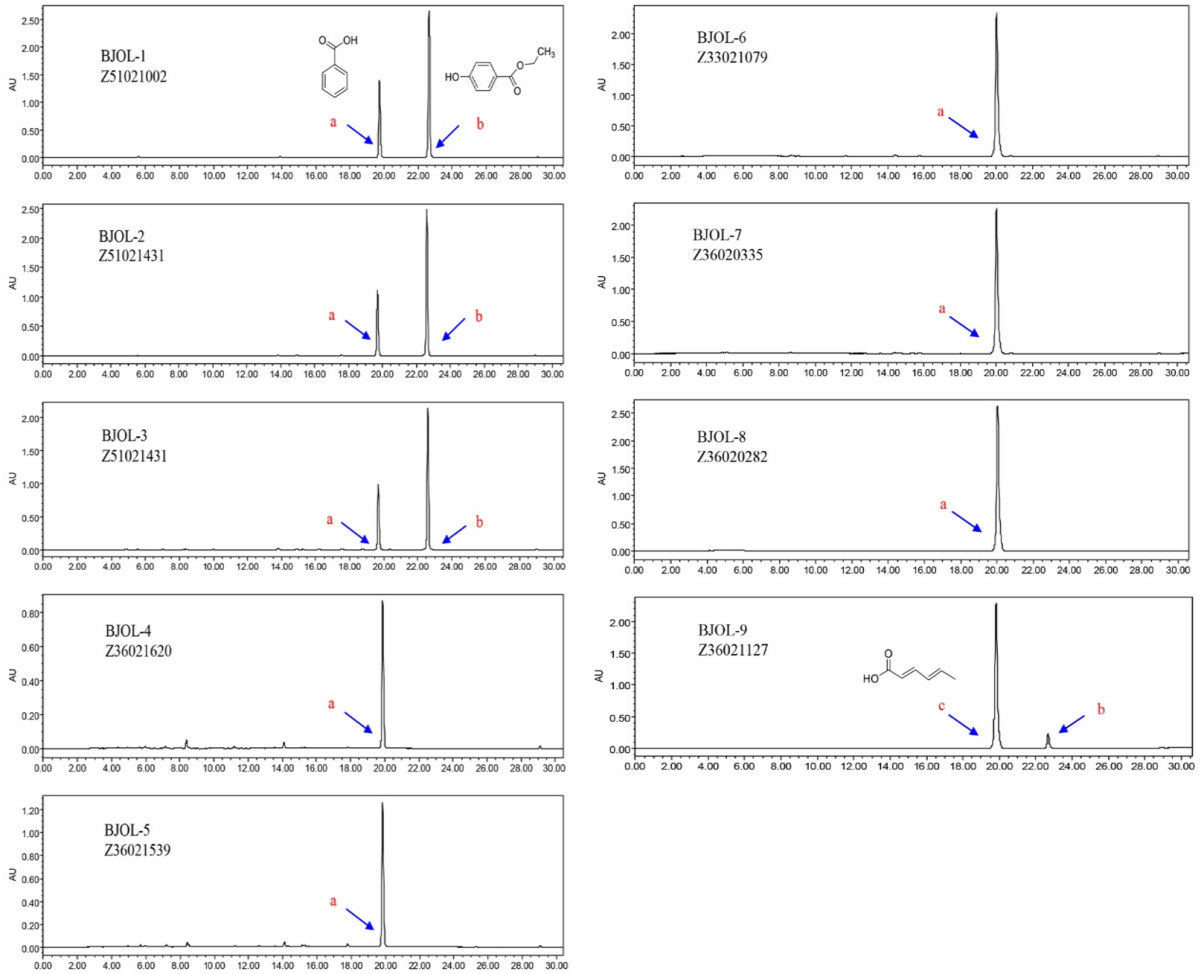
HPLC chromatogram of bamboo juice oral liquid diluted 100 times. a: benzoic acid, b: ethylparabenin, c: sorbic acid.

The quantitative results for the BJOLs available on the Chinese market are shown in Table 1. The compositions of the different oral liquids were significantly different, and the contents of the components were obviously lower than those in fresh bamboo juice. This suggested that the BJOLs had been diluted from fresh bamboo juice. Some of the overloaded peaks in the BJOLs had much greater areas than those of the other components, but these peaks did not appear in fresh bamboo juice (Fig. 4). Therefore, these peaks were separated using HPLC and identified using NMR and MS. A comparison between the NMR data and the literature indicated that these peaks corresponded to benzoic acid (a), ethylparaben (b), and sorbic acid (c). Because the contents of the three preservatives were much higher than those of main compounds of bamboo juice in BJOL, UPLC-Q-TOF-MS was unsuitable for analysis of the overloaded peaks, and quantitative analysis of these preservatives was carried out using HPLC-PAD (Table 2). The linear relationships observed are presented in Table S4 (Supplementary information). The quantitative analysis results indicated that the maximum contents of these preservatives reached 2754.25 mg L^−1^, 471.44 mg L^−1^, and 1797.48 mg L^−1^; these values were much higher than those of the main components in fresh bamboo juice. This implies that these three preservatives were artificially added; moreover, the observed levels of these preservatives are potentially harmful to the human body^34^.

**Table 2:** Additive and sugar content in fresh bamboo juice and BJOLs.

Additives and carbohydrates	Fresh bamboo juice (mg L^−1^)^a^	BJOL-1 (mg L^−1^)^a^	BJOL-2 (mg L^−1^)^a^	BJOL-2 (mg L^−1^)^a^	BJOL-4 (mg L^−1^)^a^	BJOL-5 (mg L^−1^)^a^	BJOL-6 (mg L^−1^)^a^	BJOL-7 (mg L^−1^)^a^	BJOL-8 (mg L^−1^)^a^	BJOL-9 (mg L^−1^)^a^
Benzoic acid	0	2754.25	1949.86	1570.10	1484.00	2198.40	1530.68	1497.38	1725.20	0
Ethylparaben	0	471.44	307.40	230.11	0	0	0	0	0	432.34
Sorbic acid	0	0	0	0	0	0	0	0	0	1797.48
Arabinose	64,598.73	25,968.90	4207.83	10,967.70	14,350.69	9852.98	14,019.25	6362.57	4999.10	9701.43
Glucose	126,530.52	0	29,022.95	0	3302.39	4332.53	2963.35	10,410.03	7162.85	2318.63
Xylose	6943.38	0	0	0	0	0	0	3586.61	3099.18	0
Mannose	2947.72	0	0	0	0	0	0	1283.14	0	0

^**a**^Mean ± SD, *n* = 3.

Benzoic acid was used as a cough suppressant in the nineteenth century because of its anti-microbial function^35^. However, the BJOLs from the Chinese market contained unacceptably high levels benzoic acid or other preservatives. The results could be surmised the cough relief activity of the BJOLs may be related to these preservatives.

#### Analysis of carbohydrates in bamboo juice and bamboo juice oral liquids

Four carbohydrates, namely, arabinose, glucose, xylose, and mannose, were detected by ion chromatography (Supplementary information). The calibration curve was established by analysing six different concentrations. Good linearity was established for all tested authentic samples. The linear relationships observed are presented in Table S5 (Supplementary information). The total carbohydrate content in the fresh bamboo juice reached 191,129.25 mg L^−1^, and those of the BJOLs were in the range of 10,967.70–33,230.78 mg L^−1^ (Table 2). The types and contents of carbohydrates obviously varied among the BJOLs. This would seem to further confirm that most of the BJOLs were diluted from fresh bamboo juice.

In ancient China, patients usually could not obtain enough sugar to defeat colds and bacterial infections. Considering that the contents of carbohydrates were far greater than those of other compounds in BJOLs, Chinese people in antiquity may have been able to easily acquire carbohydrates by drinking bamboo juice to boost their immunity.

## Conclusions

Twenty-six compounds were isolated from fresh bamboo juice and identified for the first time. The main chemical constituents in fresh bamboo juice were found to be compounds **12**, **13**, and **22**, with contents of 35.33 ± 0.10 mg L^−1^, 20.71 ± 0.11 mg L^−1^, and 47.15 ± 0.06 mg L^−1^. These ingredients may have potential biological activity, but they are not known to have anti-inflammation activity, which may because of the low contents. Moreover, guaiacol was not detected in bamboo juice or BJOLs.

Subsequently, qualitative and quantitative analyses of fresh bamboo juice and BJOLs showed that they had distinct compositions. This may have been due in part to differences in the bamboo material selection and production methods, but the results also indicated that most of the BJOLs had been diluted from fresh bamboo juice. Due to the differences in composition, it was difficult to determine whether fresh bamboo juice had cough relief ability.

No preservatives were found in fresh bamboo juice. However, excessively high doses of preservatives were found in the BJOLs from the Chinese market, including benzoic acid, ethylparaben, and sorbic acid. The results indicated that these preservatives were deliberately added, as confirmed by the manufacturers of the BJOLs. However, the preservative contents greatly exceeded the relevant FAO/WHO regulations (benzoic acid: 0–5 mg kg^−1^; ethylparaben: 5–10 mg kg^−1^; sorbic acid: 0–25 mg kg^−1^). Thus, the use of these BJOLs with high preservative levels as cough medicines could pose a potential hazard to human health.

Furthermore, carbohydrates were the essential components in bamboo juice and BJOLs. Thus, fresh bamboo juice could be used as natural beverage for sugar supplementation. There was no evidence to support the idea that fresh bamboo juice contains specific active trace compositions with cough relief activity, but the added preservatives in the BJOLs have anti-bacterial activity. It was speculated that the cough relief activity of the BJOLs may be related to these preservatives.

The market for bamboo juice oral liquids in China enormous, but the natural food industry has developed chaotically and lacks industrial standards for food and drug safety. Moreover, with the development of human productivity and human evolution, many traditional functional foods are no longer appropriate for humans. The evaluation of traditional functional foods and the determination of their usefulness in modern times using chemical analysis techniques represents an important research direction for the future.

## Materials and methods

### General procedures

Ultraviolet spectral were obtained on a Waters 2695 high performance liquid chromatography (HPLC) instrument with a photo diode array detector (Waters, Massachusetts, USA). Preparative HPLC was carried out on a Shimadzu LC-6AD instrument (Shimadzu, Kyoto, Japan) with a UV–Vis detector (SPD-20A), using a YMC-Pack-ODS-A column (250 × 20 mm, 5 μm) (YMC, Kyoto, Japan). The carbohydrates were measured by ion chromatography using an amperometric detector (Metrohm, Herisau, Switzerland) with a Hamilton RCX-30 column (250 × 4.6 mm, 7 μm) (Hamilton, Nevada, USA). Nuclear magnetic resonance (NMR) spectra were obtained using Bruker AV-500 spectrometers (500 MHz for ^1^H NMR and 125 MHz for ^13^C NMR) (Bruker, Zurich, Switzerland). Chemical shifts (*δ*) are given in ppm, with TMS as an internal standard, and coupling constants (*J*) are in Hz. High-resolution electrospray ionisation-mass spectrometry (HRESIMS) spectra were obtained using an Agilent 6540 ultra-performance liquid chromatography (UPLC) with high-resolution quadrupole time-of-flight (Q-TOF) mass spectrometer (Agilent, California, USA), using an RRHD Eclipse Plus C_18_ column (150 × 2.1 mm, 1.8 μm) (YMC, Kyoto, Japan)^36^.

### Materials and reagents

The liquid juice of *P. edulis* was collected in August 2018 from Tonggu County, Jiangxi Province, China. This material was identified by Professor Qirong Guo of the Co-Innovation Centre for Sustainable Forestry in Southern China, College of Forestry, Nanjing Forestry University. A voucher specimen (No. MZL180801) was deposited at the International Centre for Bamboo and Rattan.

Nine varieties of fresh bamboo juice oral liquid (BJOL) were purchased from the Chinese market; the sample identification codes used in this study and National Medical Products Administration (NMPA) numbers for the products are as follows: BJOL-1 (NO. Z51021002), BJOL-2 (NO. Z51021431, 30 mL), BJOL-3 (NO. Z51021431, 100 mL), BJOL-4 (NO. Z36021620), BJOL-5 (NO. Z36021539), BJOL-6 (NO. Z33021079), BJOL-7(NO. Z36020335), BJOL-8 (NO. 36020282), and BJOL-9 (NO. Z36021127).

Column chromatography was performed with a macroporous resin (Diaion HP-20, Mitsubishi Chemical Corp, Tokyo, Japan), RP-C_18_ (50 μm, YMC, Kyoto, Japan), and Sephadex LH-20 (Pharmacia Fine Chemicals, Uppsala, Sweden). All reagents were purchased from Beijing Chemical Works (Beijing, China) unless otherwise specified. HPLC-grade methanol (MeOH) and acetonitrile (ACN) were purchased from Fisher Scientific (Pittsburgh, PA, USA).

### Extraction and preparative separation

Liquid juice of *P. edulis* sample (100.00 kg) was concentrated under reduced pressure to obtain a residue (3.80 kg). The residue was then sequentially extracted with ethyl acetate and water-saturated *n*-butanol, yield a ethyl acetate-soluble fraction (50.48 g), and an *n*-butanol–soluble fraction (194.12 g), respectively^37^.

The *n-*butanol extract was ultrasonically dissolved in 200 mL of distilled water and subjected to gradient elution through an HP-20 type macroporous adsorption resin column. The eluting solvents were distilled water, 10% ethanol, 30% ethanol, 50% ethanol, and subsequently 95% ethanol. All the fractions were eluted separately on a LH-20 gel column using distilled water as the eluent. The samples were collected in fractions of 50 mL, and the fractions were combined according to the results of HPLC with a diode array detector (HPLC–DAD). The merged samples were then concentrated and lyophilised. Subsequent purification by C_18_ reverse-phase preparative liquid chromatography column gave compound **3** (17.9 mg), compound **7** (6.2 mg), compound **8** (5.3 mg), compound **11** (230 mg), compound **12** (248.2 mg), compound **13** (52.3 mg), compound **14** (10 mg), compound **15** (8.7 mg), compound **18** (5.3 mg), compound **20** (9.7 mg), compound **21** (94.3 mg), compound **22** (228.2 mg), compound **23** (7.8 mg), compound **24** (5.9 mg), compound **25** (17.8 mg), and compound **26** (21.9 mg).

The ethyl acetate fraction was eluted on a silica gel column with dichloromethane/methanol (from 80:1 to 1:1), and 7 fractions were obtained by HPLC analysis. The samples were collected as 5 mL fractions, and the fractions were combined according to the HPLC–DAD test results. The merged samples were then concentrated and lyophilised. Subsequent purification by C_18_ reverse-phase preparative liquid chromatography column gave compound **1** (5.3 mg), compound **2** (10.2 mg), compound **4** (6.3 mg), compound **5** (5.5 mg), compound **6** (13 mg), compound **9** (5.7 mg), compound **10** (6.4 mg), compound **16** (5.2 mg), compound **17** (5.1 mg), and compound **19** (8.3 mg).

### Quantitative analysis of compounds using UPLC-Q-TOF-MS

Quantitative analysis was carried out using UPLC-Q-TOF-MS. Compounds **1–26** were isolated and purified to 98% or higher purity and used as the reference standards. Compounds **5**, **6**, **7**, **9**, **15**, **16**, **17**, and **18** were formulated with a concentration of 1000 μg mL^−1^, compounds **1**, **3**, **4**, **8**, **10**, **19**, **20**, **23**, and **24** with a concentration of 2000 μg mL^−1^, and the remaining compounds **2, 11**, **12**, **13**, **14**, **21**, **22**, **25** and **26** with a concentration of 5000 μg mL^−1^. Each single-standard solution was precisely measured and placed in a 2-mL flask. The mixed standard solution was obtained by combining the standard solutions in chromatography-grade methanol. Acetonitrile (B)-formic acid water (A) was used as the mobile phase for elution (0–5 min, 5% B; 5–35 min, 5%-55% B). The flow rate was set to 0.25 mL min^−1^, column temperature was set at 40 ℃, and the positive ion mode was adopted. A series of concentrations of the mixed standard solution were prepared to assess the linear relationship, and each of the different concentrations of solution was injected using the stated chromatographic conditions to generate the corresponding regression equations^31^.

To prevent overloading, the 26 compounds were divided into two groups according to the response peaks of the compounds. Compounds **3**, **4**, **5**, **6**, **7**, **8**, **9**, **10**, **13**, **15**, **16**, **18**, **19**, **23** were calculated using the peak area after tenfold dilution, and the other compounds **1**, **2**, **11**, **12**, **14**, **17**, **20**, **21**, **22**, **24**, **25**, **26** were calculated using the peak area of 100-fold times dilution.

### Verification of the analytical method

UPLC-Q-TOF-MS analysis was performed to determine the limit of detection (LOD), limit of quantification (LOQ), precision, and accuracy^33^. The LOD and LOQ for the main constituents were measured by duplicate injections of standard solutions based on S/N ratios of 3 and 10, respectively. The precision of the method was studied through six replicate injections of the mixed standard solutions and was expressed as relative standard deviation (RSD). The intraday precision was studied by injecting the mixed standard solutions six times on the same day (0, 2, 4, 6, 8, and 12 h), and the inter-day precision was evaluated by injecting the mixed standard solutions six times on four consecutive days. Results are expressed as RSD. The accuracy was based on a standard addition recovery test of the method, which was performed by diluting fresh bamboo juice 10 times and 100 times and adding three concentrations of the mixed standard solution. The three replicate spiked samples were analysed using UPLC-Q-TOF-MS. Subsequently, the recovery rates of the 26 components were calculated.

### Isolation, identification, and quantitative analysis of preservatives

MeOH-formic acid water was used as the mobile phase for elution. The flow rate was set to 8 mL min^−1^ and a 250 × 20 mm C_18_ analytical column was used. The LC-6AD was used for the repeated injection of the BJOL samples from the Chinese market, and the monomers a (5 mg), b (5 mg), and c (5 mg) were obtained.

- a: ^1^H NMR (500 MHz, DMSO) *δ* 12.94 (s, 1H), 7.95 (d, *J* = 7.0 Hz, 2H), 7.60 (d, *J* = 6.5 Hz, 1H), 7.49 (t, *J* = 6.5 Hz, 2H), 3.75 (s, 1H), 3.17 (s, 1H); ^13^C NMR (125 MHz, DMSO) *δ* 167.8 (s), 133.3 (s), 129.8 (s), 128.9 (s).
- b: ^1^H NMR (500 MHz, DMSO) *δ* 10.27 (s, 1H), 7.83 (d, *J* = 8.0 Hz, 2H), 6.87 (d, *J* = 8.0 Hz, 2H), 4.22 (q, *J* = 7.0 Hz, 2H), 1.26 (t, *J* = 7.0 Hz, 3H); ^13^C NMR (125 MHz, DMSO) δ 166.18 (s), 162.55 (s), 131.92 (s), 121.28 (s), 115.84 (s), 60.56 (s), 14.71 (s).
- c: ^1^H NMR (500 MHz, DMSO) *δ* 12.09 (s, 1H), 7.14 (dd, *J* = 15.5, 9.5 Hz, 1H), 6.21 (t, overlap, 2H), 5.75 (d, *J* = 15.5 Hz, 1H), 1.79 (d, *J* = 5.0 Hz, 3H); ^13^C NMR (125 MHz, DMSO) δ 168.25 (s), 144.97 (s), 139.44 (s), 130.25 (s), 120.40 (s), 18.81 (s).

HPLC–UV analysis was conducted using a Waters 2695–2996 system. The optimal mobile phase for analysis was a binary gradient elution system consisting of solvent A (MeOH) and solvent D (water containing 0.5% acetic acid). The gradient was programmed as 5% (solvent A) to 95% (solvent A) over 30 min. The column was a YMC-PACK ODS-AQ C_18_ column. The flow rate was 1 mL min^−1^, and the column temperature was set at 25 °C. The injection volume was 10 μL. The UV response was monitored at 228 nm (λ_max_ for benzoic acid), 255 nm (λ_max_ for ethylparaben), and 259 nm (λ_max_ for sorbic acid). A series of mixed standard working solutions of benzoic acid, ethylparaben, and sorbic acid were sequentially injected into the liquid chromatograph to determine the corresponding peak areas. The standard curve was constructed using the concentration as the abscissa and the peak area as the ordinate. The samples BJOL-1 to BJOL-9 were injected into the liquid chromatograph to obtain the peak area and concentration of benzoic acid, ethylparaben, or sorbic acid in the solutions according to the standard curve^38,39^.

### Detection of carbohydrates in bamboo juice oral liquids

Analysis of the composition of polysaccharides was based on the sulfuric acid acidolysis method with some modifications^33^. The fresh bamboo juice and BJOLs were diluted 1000 times as test sample solutions. The sample solutions (10 μL) were treated with 72% H_2_SO_4_ and ultrapure water in a brown bottle at 105 °C for 2.0 h. Once cooled, the acidolysis solution was neutralised to pH 7.0 using 2.0 mM NaOH, and a portion of the sample (5 mL) was added to 10 mL of ultrapure water and then filtered using a membrane filter with a pore size of 0.22 mm. The filtrate (10 mL) was then tested. The detection of carbohydrates (i.e., arabinose, glucose, xylose, and mannose) and analyses of the carbohydrates were conducted using a mobile phase consisting of ultrapure water with 2.0 mM NaOH and 0.5 mM NaAc at a rate of 1 mL min^−1^ for 60 min. The injection volume was 2 mL. Five standard monosaccharide stock solutions (0.5 mg mL^−1^) were prepared by mixing in a 10-mL volumetric flask and diluted to six different concentrations (1, 5, 25, 50, 150, and 300 mg L^−1^) with ultrapure water to examine the linear relationship^40,41^.

### Data processing and statistical analysis

The test results were expressed as mean ± SD (n = 3). The software used to analyse the test data was SPSS 16.0, and analysis of variance was used for the significance study.

## Supplementary information

Supplementary Information.
