# Supplementary material for: Identification and quantitation of the actual active components in bamboo juice and its oral liquid by NMR and UPLC-Q-TOF-MS
Source: Sci Rep. 2020 Nov 12;10:19664. doi: 10.1038/s41598-020-76897-3 (PMC7661497; doi:10.1038/s41598-020-76897-3)
Supplement: Supplementary file 1 — Supplementary Information. [file 41598_2020_76897_MOESM1_ESM.pdf]

Identification and quantitation of the actual active components in bamboo juice and its oral liquid by NMR and UPLC-Q-TOF-MS

Quan Gao[1,‡], Detao Wang[2,‡], Siyue Shao[2], Yingying Xue[2], Yingfang Zhang[2], Chen Chen[2], Feng Tang[2], Jia Sun[2,‡,*], Yansu Li[3,*], Qirong Guo[4]

1 School of Plant Protection, Anhui Agricultural University, Hefei 230036, China

2 State Forestry Administration Key Open Laboratory, International Centre for Bamboo and Rattan, Beijing 100102, China

3 Institute of Vegetables and Flowers, the Chinese Academy of Agricultural Sciences, Beijing 100081, China

4 College of Forestry, Nanjing Forestry University, Nanjing 210037, China

* Corresponding Authors.

sunjia@icbr.ac.cn (J, Sun) and liyansu@caas.cn (YS, Li).

Tel.: +86 010-84789912; Fax: +86 010-84789821.

Supplementary information

NMR and MS data for compounds **1-26**.

Table S1. Limits of detection, accuracy, and precision of compounds **1-26**.

Table S2. Accuracy test results of compounds **1-26**.

Table S3. Results of liner relationship of compounds **1-26**.

Table S4. Results of liner relationship of additive contents.

Table S5. Results of liner relationship of carbohydrate.

Fig. S1. Compounds **1-26** purification verification chromatography.

Fig. S2. HPLC chromatogram of three preservatives.

Fig. S3. Ion chromatography of carbohydrate.

NMR and MS data for compounds 1-26

Compound **1**

ESI-MS *m/z*: 155.0703 $[M+H]^+$, $C_8H_{10}O_3$; ^{1}H NMR (500 MHz, DMSO) δ 6.87 (s, 1H, H-5), 6.69 (s, 2H, H-2,6), 4.36 (s, 2H, H-7), 3.75 (3-OCH_3); ^{13}C NMR (125 MHz, DMSO) δ 147.7 (C-3), 145.7 (C-4), 133.9 (C-1), 119.5 (C-6), 115.4 (C-5), 111.5 (C-2), 63.4 (C-7), 55.9 (4-OCH_3).

Compound **2**

ESI-MS *m/z*: 197.0802 $[M+H]^+$, $C_{10}H_{12}O_4$; ^{1}H NMR (500 MHz, DMSO) δ 7.51 (d, J = 8.0 Hz, 1H, H-2), 7.44 (s, 1H, H-6), 6.86 (d, J = 8.0 Hz, 1H, H-3), 3.82 (s, 3H, 5-OCH_3), 3.75 (t, J = 6.0 Hz, 2H, H-3′), 3.05 (t, J = 6.0 Hz, 2H, H-2′); ^{13}C NMR (125 MHz, DMSO) δ 197.67 (C-1′), 152.2 (C-4), 147.9 (C-5), 129.3 (C-1), 123.5 (C-2), 115.4 (C-3), 111.6 (C-6), 57.7 (C-3′), 56.1 (5-OCH3), 41.4 (C-2′).

Compound **3**

ESI-MS *m/z*: 237.0719 $[M+Na]^+$, $C_{10}H_{14}O_5$; ^{1}H NMR (500 MHz, DMSO) δ 6.89 (s, 1H, H-2), 6.70 (s, 2H, H-5,6), 4.89 (s, 1H, H-7), 4.43 (d, J = 11.0 Hz, 1H, H-8), 3.74 (s, 3H, 3-OCH_3), 3.44 (s, 1H, H-9), 3.33 (d, J = 11.0 Hz,1H, H-9); ^{13}C NMR (125 MHz, DMSO) δ 147.5 (C-3), 145.8 (C-4), 134.8 (C-1), 119.6 (C-6), 115.2 (C-5), 111.5 (C-2), 76.4 (C-7), 73.4 (C-8), 63.1 (C-9), 56.1 (3-OCH_3).

Compound **4**

ESI-MS *m/z*: 211.0960 $[M+H]^+$, $C_{11}H_{14}O_4$; ^{1}H NMR (500 MHz, DMSO) δ 9.75 (s, 1H, CHO), 7.19 (s, 2H, H-2, 6), 3.84 (s, 6H, 3, 5-OCH_3); ^{13}C NMR (125 MHz, DMSO) δ 191.4 (CHO), 148.7 (C-3, 5), 143.3 (C-4), 127.2 (C-1), 107.6 (C-2, 6), 56.5 (OCH_3).

Compound **5**

ESI-MS *m/z*: 199.0596 $[M+H]^+$, $C_9H_{10}O_5$; ^{1}H NMR (500 MHz, DMSO) δ 7.20 (s, 2H, H-3,5), 3.79 (2,6-OCH_3); ^{13}C NMR (125 MHz, DMSO) δ 166.6 (COOH), 148.1 (C-2,6), 143.6 (1-OH), 127.8 (C-4), 107.3 (C-3,5), 56.5 (2×OCH_3).

Compound **6**

ESI-MS *m/z*: 183.0648 $[M+H]^+$, $C_9H_{10}O_4$; ^{1}H NMR (500 MHz, DMSO) δ 9.75 (s, 1H, CHO), 7.19 (s, 2H, H-2,6), 3.84 (s, 6H, 3,5-OCH_3); ^{13}C NMR (125 MHz, DMSO) δ 191.4 (CHO), 148.7 (C-3,5), 143.3 (C-4), 127.2 (C-1), 107.6 (C-2,6).

Compound **7**

ESI-MS *m/z*: 165.0535 $[M+H]^+$, $C_9H_8O_3$; ^{1}H NMR (500 MHz, DMSO) δ 11.01 (s, 1H, COOH), 7.50 (d, J = 7.0 Hz, 2H, H-2, 6), 7.45 (s, 1H, H-7), 6.75 (t, J = 18.5 Hz, 2H, H-3, 5), 6.29 (d, J = 15.5 Hz, 1H, H-

8); ^{13}C NMR (125 MHz, DMSO) δ 168.6 (C-COOH), 159.9 (C-4), 144.3 (C-7), 130.4 (C-2, 6), 125.8 (C-1), 116.2 (C-3, 5).

Compound **8**

ESI-MS *m/z*: 335.1094 $[M+Na]^+$, $C_{15}H_{20}O_7$; ^1H NMR (500 MHz, DMSO) δ 7.07 (d, 2H, H-2, 6), 6.75 (s, 1H, H-7), 6.43 (d, J = 9.5 Hz, 2H, H-3,5), 5.64 (s, 1H, H-8), 4.33 (s, 1H, H-1′), 4.19 (s, 1H, H-9), 4.04 (s, 1H, H-9), 3.51 (s, 1H, H-6′), 3.34 (m, 4H, H-2, 3, 4,5); ^{13}C NMR (125 MHz, DMSO) δ 157.2 (C-4), 130.8 (C-1), 130.5 (C-7), 127.6 (C-2, 6), 126.6 (C-8), 115.6 (C-3, 5), 102.8 (C-1′), 77.3 (C-5′), 77.2 (C-3′), 73.8 (C-2′), 70.5 (C-4′), 65.9 (C-9), 61.5 (C-6′).

Compound **9**

ESI-MS *m/z*: 179.0698 $[M+H]^+$, $C_{10}H_{10}O_3$; ^1H NMR (500 MHz, DMSO) δ 9.57 (d, J = 7.5 Hz, 1H, H-CHO), 7.58 (d, J = 15.5 Hz, 1H, H-7), 7.32 (s, 1H, H-6), 7.16 (d, J = 7.5 Hz, 1H, H-8), 6.81 (d, J = 8.0 Hz, 1H, H-3), 6.71 (dd, J = 15.5, 8.0 Hz, 1H, H-2), 3.82 (s, 3H, OCH_3); ^{13}C NMR (125 MHz, DMSO) δ 194.3 (CHO), 154.6 (C-5), 148.7 (C-4), 128.6 (C-8), 127.1 (C-7), 125.7 (C-1), 124.7 (C-2), 116.3 (C-3), 111.9 (C-6), 56.2 (OCH_3).

Compound **10**

ESI-MS *m/z*: 181.0847 $[M+H]^+$, $C_{10}H_{12}O_3$; ^1H NMR (500 MHz, DMSO) δ 6.99 (s, 1H, H-6), 6.79 (d, J = 7.5 Hz, 1H, H-2), 6.70 (d, J = 7.5 Hz, 1H, H-5), 6.41 (d, J = 15.5 Hz, 1H, H-7), 6.24 – 6.09 (m, 1H, H-8), 4.07 (s, 2H, H-9), 3.78 (s, 3H, H-OCH_3); ^{13}C NMR (125 MHz, DMSO) δ 148.2 (C-3), 146.7 (C-4), 129.4 (C-1), 128.9 (C-7), 127.9 (C-8), 119.9 (C-6), 115.9 (C-5), 110.3 (C-2), 62.2 (C-9), 56.1 (3-OCH_3).

Compound **11**

ESI-MS m/z: 273.2039 $[M+H]^+$, $C_{12}H_{16}O_7$; ^1H NMR (500 MHz, DMSO) δ 6.86 (d, J = 8.5 Hz, 2H, H-3, 5), 6.65 (d, J = 8.5 Hz, 2H, H-2, 6); ^{13}C NMR (125 MHz, DMSO) δ 152.7 (C-4), 150.8 (C-1), 118.2 (C-3, 5), 115.9 (C-2, 4), 102.3 (C-1′), 77.4 (C-5′), 77.1 (C-3′), 73.8 (C-2′), 70.3 (C-4′), 61.3 (C-6′).

Compound **12**

ESI-MS m/z: 325.0890 $[M+Na]^+$, $C_{13}H_{18}O_8$; ^1H NMR (500 MHz, DMSO) δ 6.68 (s, 1H,H-5), 6.65 (d, J = 8.5 Hz, 1H,H-6), 6.45 (d, J = 8.0 Hz, 1H,H-3), 5.22 (d, J = 3.0 Hz, 1H); ^{13}C NMR (125 MHz, DMSO) δ 151.2 (C-4), 148.3 (C-2), 141.8 (C-1), 115.6 (C-6), 108.5 (C-5), 103.1 (C-3), 102.2 (C-1′), 77.5 (C-5′), 77.2 (C-3′), 73.8 (C-2′), 70.4 (C-4′), 61.3 (C-6′).

Compound **13**

ESI-MS *m/z*: 355.0976 $[M+Na]^+$, $C_{14}H_{20}O_9$; ^1H NMR (500 MHz, DMSO) δ 7.84 (s, 1H, H-4), 6.38 (s, 2H, H-2,6), 5.22 (d, J = 3.5 Hz, 1H), 5.06 (s, 1H), 5.00 (d, J = 4.0 Hz, 1H), 4.68 (d, J = 7.0 Hz, 1H), 4.61 (s, 1H), 3.71 (2×OCH_3), 3.52 – 3.37 (m, 1H), 3.27 (dd, J = 19.5, 11.8 Hz, 4H), 3.11 (s, 1H); ^{13}C NMR (125 MHz, DMSO) δ 150.7 (C-3,5), 148.6 (C-1), 130.9 (C-4), 102.1 (C-1′), 95.6 (C-2,6), 77.6 (C-5′), 77.3 (C-3′), 73.8 (C-2′), 70.6 (C-4′), 61.4 (C-6′).

Compound **14**

ESI-MS *m/z*: 351.1048 $[M+Na]^+$, $C_{15}H_{20}O_8$; ^1H NMR (500 MHz, DMSO) δ 7.84 (d, J = 7.5 Hz, 2H, H-2, 6), 6.82 (d, J = 7.5 Hz, 2H, H-3, 5), 4.18 (s, 1H, H-9), 4.08 (s, 1H, H-9), 3.20 (s, 1H, H-8), 3.08 (s, 1H, H-8); ^{13}C NMR (125 MHz, DMSO) δ 196.7 (C-7), 163.3 (C-4), 131.1 (C-2, 6), 128.5 (C-1), 115.8 (C-3, 5), 103.6 (C-1′), 77.4 (C-5′), 77.3 (C-3′), 73.9 (C-2′), 70.6 (C-4′), 65.1 (C-9), 61.6 (C-6′), 40.8 (C-8).

Compound **15**

ESI-MS *m/z*: 381.1186 $[M+Na]^+$, $C_{16}H_{22}O_9$; ^1H NMR (500 MHz, DMSO) δ 7.50 (d, J = 6.5 Hz, 1H, H-6), 7.41 (s, 1H, H-2), 6.99 (d, 1H, H-5), 3.80 (s, 3H, 3-OCH_3); ^{13}C NMR (125 MHz, DMSO) δ 196.4 (C-7), 148.4 (C-4), 148.2 (C-3), 147.9(C-1), 123.9 (C-6), 115.6 (C-5), 111.4 (C-2), 103.6 (C-1′), 77.3 (C-5′), 77.2 (C-3′), 73.9 (C-2′), 70.6 (C-4′), 65.3 (C-6′), 61.5 (C-9), 55.9 (C-8).

Compound **16**

ESI-MS *m/z*: 357.1329 $[M+H]^+$, $C_{20}H_{20}O_6$; ^1H NMR (500 MHz, DMSO) δ 9.61 (CHO), 7.65 (d, J = 16 Hz, 1H, H-7′), 7.31 (s, 1H, H-2′), 6.93 (s, 1H, H-5), 6.79 (d, J = 12.5 Hz, 1H, H-8′), 5.56 (d, J = 6.5 Hz, 1H, H-7), 5.06 (s, 1H), 4.08 (s, 1H), 3.84 (s, 3H, OCH_3), 3.75 (s, 3H, OCH_3); ^{13}C NMR (125 MHz, DMSO) δ 194.64 (CHO), 154.7 (C-7′), 151.2 (C-4′), 148.1 (C-3), 147.1 (C-4), 144.6 (C-3′), 132.2 (C-1), 130.7 (C-1′), 128.2 (C-5′), 126.6 (C-8′), 119.5 (C-6), 119.2 (C-6′), 115.9 (C-5), 112.9 (C-2′), 110.9 (C-2), 88.7 (C-7), 63.2 (C-9), 56.4 (C-8), 56.1 (OCH_3), 52.9 (OCH_3).

Compound **17**

ESI-MS *m/z*: 387.1432 $[M+H]^+$, $C_{21}H_{22}O_7$; ^1H NMR (500 MHz, DMSO) δ 9.60 (CHO), 7.66 (d, J = 16 Hz, 1H, H-7′), 7.33 (s, 2H), 6.79 (dd, J = 16, 8.0 Hz, 2H), 6.64 (s, 2H), 6.61 (s, 1H), 5.55 (d, J = 7.0 Hz, 1H), 3.85 (s, 3H), 3.76 (s, 2H), 3.73 (s, 6H), 3.56 (d, J = 6.5 Hz, 1H), 3.17 (s, 3H); ^{13}C NMR (125 MHz, DMSO) δ 194.5 (CHO), 154.4 (C-7′), 151.2 (C-4′), 148.5 (C-3, 5), 144.6 (C-4), 130.8 (C-3′), 128.2 (C-1), 126.6 (C-1′), 119.4 (C-5′), 113.1 (C-8′), 104.4 (C-2, 6), 88.9 (C-6′), 62.9 (C-7), 56.5 (C-9), 56.4 (C-8), 52.8 (2×OCH_3), 49.1 (OCH_3).

Compound **18**

ESI-MS *m/z*: 169.0479 [M+H]$^+$, $C_8H_8O_4$; ^{1}H NMR (500 MHz, DMSO) δ 5.97 (s, 2H, H-3, 5), 3.75 (s, 6H, OCH_3); ^{13}C NMR (125 MHz, DMSO) δ 187.5 (C-4), 176.5 (C-1), 157.7 (C-2, 6), 107.5 (C-3, 5), 56.9 (OCH_3).

Compound **19**

ESI-MS *m/z*: 127.0388 [M+H]$^+$, $C_6H_6O_3$; ^{1}H NMR (500 MHz, DMSO) δ 7.48 (d, J = 2.5 Hz, 1H, H-4), 6.60 (d, J = 2.5 Hz, 1H, H-4), 4.51 (s, 2H, H-2); ^{13}C NMR (125 MHz, DMSO) δ 178.40 (CHO), 162.6 (C-2), 152.2 (C-5), 124.8 (C-4), 110.1 (C-3), 56.4 (C-2).

Compound **20**

ESI-MS *m/z*: 268.1039 [M+H]$^+$, $C_{10}H_{13}N_5O_4$; ^{1}H NMR (500 MHz, DMSO) δ 8.34 (s, 1H, H-2), 8.13 (s, 1H, H-8), 7.32 (s, 2H, H-6), 5.97 (s, 1H. H-1′), 5.88 (d, J = 5.5 Hz, 1H, H-2′), 5.42 (d, 1H, H-3′), 5.17 (d, J = 2.5 Hz, 1H, H-4′); ^{13}C NMR (125 MHz, DMSO) δ 156.60 (C-6), 152.8 (C-2), 149.5 (C-4), 140.3 (C-8), 119.8 (C-5), 88.3 (C-1′), 86.3 (C-4′), 73.9 (C-2′), 71.1 (C-3′), 62.1 (C-5′).

Compound **21**

ESI-MS *m/z*: 325.0890 [M+Na]$^+$, $C_{13}H_{18}O_8$; ^{1}H NMR (500 MHz, DMSO) δ 6.89 (d, J = 8.5 Hz, 1H, H-3), 6.40 (s, 1H, H-6), 6.23 (d, J = 8.0 Hz, 1H, H-4), 4.67 (d, J = 6.0 Hz, 1H, H-1′), 4.47 (s, 1H), 3.64 (s, 1H), 3.20 (s, 4H); ^{13}C NMR (125 MHz, DMSO) δ 153.2 (C-5), 150.4 (C-1), 139.9 (C-2), 117.9 (C-3), 106.5 (C-4), 102.1 (C-1″), 77.4 (C-5″), 77.3 (C-3″), 73.8 (C-2″), 70.3 (C-4″), 61.3 (C-6″).

Compound **22**

ESI-MS *m/z*: 605.2199 [M+Na]$^+$, $C_{28}H_{38}O_{13}$; ^{1}H NMR (500 MHz, DMSO) δ 6.43 (s, 1H, H-6), 6.23 (s, 2H, H-2′, 6′), 4.06 (d, 1H, H-7′), 3.64 (s, 3H, OCH_3), 3.53 (s, 6H, 2×OCH_3), 3.39 (m, 1H, H-9), 3.17 (s, 3H, OCH_3), 3.04 (d, 1H, H-9), 2.96 (d, 2H, H-9′), 2.52 (d, J = 12.0 Hz, 1H, H-7), 2.40 (d, J = 12.0 Hz, 1H, H-7), 1.86 (s, 1H, H-8′), 1.39 (s, 1H, H-8); ^{13}C NMR (125 MHz, DMSO) δ 148.6 (C-3′, 5′), 148.1 (C-3), 147.6 (C-5), 138.6 (C-4), 138.4 (C-1′), 134.5 (C-4′), 129.6 (C-1), 126.1 (C-2), 107.9 (C-6), 107.1 (C-2′, 6′), 104.5 (C-1″), 78.1 (C-5″), 78.0 (C-3″), 74.7 (C-2″), 71.3 (C-4″), 70.8 (C-6″), 65.2 (C-9), 62.3 (C-9′), 60.0 (3- OCH_3), 57.2 (C-3′, 5′- OCH_3), 56.8 (5- OCH_3), 45.5 (C-8′), 41.7 (C-7′), 41.4 (C-8), 33.5 (C-7).

Compound **23**

ESI-MS *m/z*: 575.209 [M+Na]$^+$, $C_{27}H_{36}O_{12}$; ^{1}H NMR (500 MHz, DMSO) δ 6.60 (s, 1H, H-2), 6.43 (s, 2H, H-2′, 6′), 6.09 (s, 1H, H-5), 4.07 (d, J = 10.9 Hz, 1H, H-7′), 3.93 (d, J = 8.0 Hz, 1H, H-1″), 3.92 (d,

1H, H-9′), 3.59 (d, 1H, H-9), 3.47 (d, 1H, H-9), 3.14 – 2.95 (m, 4H), 2.92 (d, *J* = 8.0 Hz, 1H, H-9′), 2.73 (d, *J* = 7.5 Hz, 2H, H-7), 1.92 (s, 1H, H-8′), 1.72 (s, 1H, H-8); ^{13}C NMR (125 MHz, DMSO) δ 148.3 (C-3′, 5′), 146.1 (C-3), 144.5 (C-4), 136.3 (C-1′), 134.1 (C-4′), 133.1 (C-6), 127.5 (C-1), 116.6 (C-5), 112.3 (C-2), 107.1 (C-6′), 104.8 (C-1″), 77.4 (C-5″), 77.3 (C-3″), 74.1 (C-2″), 70.5 (C-4″), 68.1 (C-9′), 63.2 (C-9), 61.5 (C-6″), 56.5 (C-3′, 5′-OCH_3), 55.9 (C-3), 46.7 (C-7′), 46.6 (C-8′), 44.4 (C-8), 32.6 (C-7).

Compound **24**

ESI-MS *m/z*: 605.2194 $[M+Na]^+$, $C_{28}H_{38}O_{13}$; ^{1}H NMR (500 MHz, DMSO) δ 6.55 (s, 1H, H-6), 6.28 (s, 2H, H-2′, 6′), 4.19 (d, *J* = 5.0 Hz, H-7′), 4.07 (d, *J* = 7.5 Hz, H-9′), 3.76 (s, 3H, OCH_3), 3.63 (s, 6H, 2×OCH_3), 3.27 (s, 3H, OCH_3), 3.14 – 2.95 (m, 5H), 2.01 (s, 1H, H-8′), 1.47 (s, 1H, H-8); ^{13}C NMR (125 MHz, DMSO) δ 148.1 (C-3′, 5′), 147.4 (C-3), 146.9 (C-5), 138.1 (C-4), 137.7 (C-1′), 133.9 (C-4′), 129.1 (C-1), 125.3 (C-2), 107.1 (C-6), 106.4 (C-2′, 6′), 103.6 (C-1″), 77.5 (C-5″), 77.3 (C-3″), 74.1 (C-2″), 70.5 (C-4″), 64.6 (C-9), 61.6 (C-9′), 59.5 (3-OCH_3), 56.6 (C-3′, 5′-OCH_3), 56.2 (5-OCH_3) 44.8 (C-8′), 41.23 (C-7′), 40.9 (C-8), 32.8 (C-7).

Compound **25**

ESI-MS *m/z*: 605.2189 $[M+Na]^+$, $C_{28}H_{38}O_{13}$; ^{1}H NMR (500 MHz, DMSO) δ: 6.59 (2H, s, H-2′,6′), 6.44 (2H, s, H-2, 6), 4.87 (1H, d, *J*=7.0Hz, H-1″), 4.72 (1H, d, *J*=6.0Hz, H-7′), 3.90 (1H, dd, *J*=8.0Hz, H-9), 3.75 (1H, m, H-9′), 3.74 (6H, s, 3′,5′-OCH3), 3.72 (6H, s, 3,5-OCH_3), 3.60 (1H, dd, *J*=7.5, 8.0Hz, H-9), 3.58 (1H, m, H-6″), 3.51 (1H, m, H-9′), 3.40 (1H, m, H-6′), 3.18 (1H, m, H-3″), 3.11 (1H, m, H-2″), 3.09 (1H, m, H-5″), 3.03 (1H, m, H-4″), 2.83 (1H, dd, *J*=4.5, 13.5 Hz, H-7), 2.60 (1H, m, H-8), 2.41 (1H, dd, *J*=11.5, 13.5Hz, H-7), 2.23 (1H, m, H-8′); ^{13}C NMR (125MHz, DMSO) δ: 153 (C-3′,5′), 148.4 (C-3,5), 140.1 (C-1′), 134 (C-4′), 133.8 (C-4), 131.4 (C-1), 106.3 (C-2,6), 104.3 (C-2′,6′), 103.2 (C-1″), 82.3 (C-7′), 77.7 (C-5″), 77.0 (C-3″), 74.7 (C-2″), 72.5 (C-9), 70.4 (C-4″), 61.4 (C-6″), 59.2 (C-9′), 56.9 (C-3′,5′-OCH3), 56.4 (3,5-OCH3), 52.9 (C-8′), 42.5 (C-8), 33.2 (C-7).

Compound **26**

ESI-MS *m/z*: 603.2039 $[M+Na]^+$, $C_{28}H_{36}O_{13}$; ^{1}H NMR (500 MHz, DMSO) δ 6.66 (s, 2H, H-2, 6), 6.60 (s, 2H, H-2′, 6′), 4.66 (d, *J* = 4.5 Hz, 1H, H-7′), 4.61 (d, *J* = 4.5 Hz, 1H, H-7), 4.33 (s, 1H), 4.18 (dd, *J* = 7.0, 4.8 Hz, 2H, H-9, 9′), 3.79 (dd, *J* = 7.0, 3.9 Hz, 2H, H-9, 9′), 3.21 – 3.17 (m, 4H); ^{13}C NMR (125 MHz, DMSO) δ 153.1 (C-3, 5), 148.4 (C-3′, 6′) 137.7 (C-4), 135.3 (C-4′), 134.1 (C-1′), 131.8 (C-1), 104.6 (C-2′, 6′), 104.1 (C-2, 6), 103.1 (C-1″), 85.9 (C-7′), 85.6 (C-7), 77.75 (C-5″), 77.01 (C-3″), 74.65

(C-2″), 71.75 (C-4″), 71.7 (C-9′), 70.4 (C-9), 61.39 (C-6″), 56.9 (OCH_3), 56.5 (OCH_3), 54.2 (C-8′), 54.1 (C-8).

Table S1. Limits of detection, accuracy, and precision of compounds **1-26**

Compound	LOD (mg/L)	LOQ (mg/L)	Accuracy precision (RSD%, n=6)	Intraday precision (RSD%, n=6)	Interday precision (RSD%, n=6)
**1**	2×10^{-2}	0.35×10^{-1}	0.92	0.83	1.44
**2**	4×10^{-4}	0.12×10^{-2}	1.22	1.32	2.42
**3**	1.4×10^{-3}	0.23×10^{-2}	0.63	1.24	0.94
**4**	1×10^{-2}	0.05	1.28	0.75	1.75
**5**	2×10^{-2}	0.05	0.81	1.24	2.52
**6**	4.8×10^{-3}	0.12×10^{-1}	0.51	0.41	1.64
**7**	5×10^{-1}	1.03	0.95	0.81	1.47
**8**	7×10^{-3}	2.21×10^{-2}	1.46	1.43	1.52
**9**	1.1×10^{-2}	0.51×10^{-1}	2.35	1.69	1.81
**10**	2.83×10^{-1}	3.25×10^{-1}	1.04	1.41	1.18
**11**	0.58×10^{-2}	2.12×10^{-2}	1.03	1.75	1.21
**12**	3×10^{-2}	0.06	0.91	1.02	1.46
**13**	0.36×10^{-2}	0.02	1.01	2.03	1.56
**14**	0.05×10^{-2}	0.01×10^{-1}	1.15	0.81	1.19
**15**	0.12×10^{-2}	0.24×10^{-2}	0.56	0.87	2.13
**16**	0.04×10^{-2}	0.08×10^{-2}	0.95	0.87	1.21
**17**	0.13×10^{-2}	0.37×10^{-2}	1.13	1.21	1.77
**18**	0.14×10^{-2}	0.23×10^{-2}	0.61	1.71	0.53
**19**	0.68×10^{-2}	0.28×10^{-1}	1.49	1.61	1.71
**20**	0.13×10^{-2}	0.22×10^{-2}	1.32	1.42	1.56
**21**	0.02×10^{-1}	0.01	0.51	0.67	0.83
**22**	0.06×10^{-1}	0.12×10^{-1}	1.03	1.35	1.91
**23**	0.08×10^{-1}	0.32×10^{-2}	1.39	1.32	1.87
**24**	0.08×10^{-1}	0.28×10^{-1}	1.21	1.98	2.17
**25**	0.52×10^{-2}	1.24×10^{-2}	1.46	1.72	0.98
**26**	0.09×10^{-2}	0.15×10^{-2}	1.42	1.54	2.02

Table S2. Accuracy test results of compounds **1-26**

No.	Recovery (%)	RSD (%)	Recovery (%)	RSD (%)	Recovery (%)	RSD (%)
**1**	103.37	1.29	102.89	2.23	95.11	1.97
**2**	104.33	1.25	115.28	0.88	118.71	3.76
**3**	87.6	0.52	88.96	1.77	88.75	1.99
**4**	92.67	2.17	89.21	1.84	88.35	1.77
**5**	92.13	1.96	97.3	2.83	97.33	2.99
**6**	89.45	0.59	92.33	0.85	89.46	0.61
**7**	94.64	1.41	90.37	0.9	90.32	2.92
**8**	93.59	0.89	91.39	2.66	87.42	2.07
**9**	90.45	2.64	96.95	2.37	92.24	0.57
**10**	94.07	1.41	90.71	1.55	90.04	2.28
**11**	107.24	1.72	111.75	2.67	91.25	2.71
**12**	108.67	1.41	94.39	2.64	90.63	0.86
**13**	103.88	1.65	90.61	2.33	91.22	2.15
**14**	119.33	0.97	107.5	0.66	91.63	1.85
**15**	93.47	1.31	92.08	2.22	88.82	2.99
**16**	89.16	1.07	92.36	1.27	88.68	0.24
**17**	99.55	1.14	96.86	2.05	92.21	1.29
**18**	90.64	2.11	91.41	1.98	91.11	2.42
**19**	88.87	1.71	90.72	0.81	91.79	2.23
**20**	104.54	1.41	98.98	2.25	97.39	2.23
**21**	98.23	0.75	93.24	1.34	90.22	0.87
**22**	106.81	0.97	114.23	4.52	99.78	2.18
**23**	90.08	1.55	92.18	0.44	88.19	1.76
**24**	105.01	0.54	95.56	1.99	95.24	2.65
**25**	98.23	1.73	95.86	2.54	96.9	2.52
**26**	110.86	1.24	114.69	2.61	112.61	2.69

Table S3. Results of liner relationship of compounds **1-26**

No.	Molecular mass	Linear equation	R²	Linear range	Content
**1**	155.0703$[M+H]^+$	y = 36499x+ 28848	R^2 = 0.9978	1.2×10^{-1}-10	2.31
**2**	197.0802$[M+H]^+$	y = 2×10^7x + 10824	R^2 = 0.9991	2.3×10^{-3}-0.54	0.43
**3**	237.0719$[M+Na]^+$	y = 4×10^6x - 1298.3	R^2 = 0.9997	2.8×10^{-3}-0.28	0.15
**4**	211.0960$[M+H]^+$	y = 55489x + 65521	R^2 = 0.9996	1.1×10^{-1}-27.5	2.13
**5**	199.0596$[M+H]^+$	y = 103029x + 13044	R^2 = 0.9991	1.3×10^{-1}-12.5	4.11
**6**	183.0648$[M+H]^+$	y = 382839x + 2590.5	R^2 = 0.9985	2.4×10^{-2}-6	1.43
**7**	165.0535$[M+H]^+$	y = 28205x + 79386	R^2 = 0.9990	5-80	7.34
**8**	335.1094$[M+Na]^+$	y = 484453x + 9440.8	R^2 = 0.9998	3.5×10^{-2}-3.5	1.68
**9**	179.0698$[M+H]^+$	y = 697754x + 78221	R^2 = 0.9991	5.5×10^{-2}-5.5	7.21
**10**	181.0847$[M+H]^+$	y = 31273x + 2771.4	R^2 = 0.9992	2.5-40	5.03
**11**	295.0758$[M+Na]^+$	y = 346037x + 7350.2	R^2 = 0.9995	2.32×10^{-2}-1.16	15.21
**12**	325.0890$[M+Na]^+$	y = 586432x + 220829	R^2 = 0.9992	12×10^{-2}-15	35.37
**13**	355.0976$[M+Na]^+$	y = 58216x + 5537.6	R^2 = 0.9990	2.8×10^{-2}-3.5	20.58
**14**	351.1048$[M+Na]^+$	y = 2×10^7x + 54590	R^2 = 0.9991	2×10^{-2}-0.2	12.73
**15**	381.1186$[M+Na]^+$	y = 4×10^6x - 26825	R^2 = 0.9998	4.8×10^{-3}-0.6	7.34
**16**	357.1329$[M+H]^+$	y = 8×10^6x + 67039	R^2 = 0.9982	3.2×10^{-3}-0.4	1.07
**17**	387.1432$[M+H]^+$	y = 5×10^6x + 13639	R^2 = 0.9992	5.2×10^{-3}-0.26	1.72
**18**	169.0479$[M+H]^+$	y = 2×10^6x + 22611	R^2 = 0.9996	7.2×10^{-3}-0.9	1.71
**19**	127.0388$[M+H]^+$	y = 516891x + 49813	R^2 = 0.9990	3.4×10^{-2}-3.4	2.96
**20**	268.1039$[M+H]^+$	y = 1×10^8x + 154801	R^2 = 0.9994	2.6×10^{-3}-0.13	0.62
**21**	355.0976[M+Na]+	y = 58216x + 5537.6	R^2 = 0.9990	2.8×10-2-3.5	16.58
**22**	605.2199$[M+Na]^+$	y = 1×10^6x + 30.324	R^2 = 0.9999	2.4×10^{-2}-3	47.13
**23**	575.2091$[M+Na]^+$	y = 230030x + 6456.1	R^2 = 0.9989	8×10^{-2}-8	17.74
**24**	605.2194$[M+Na]^+$	y = 521054x - 15576	R^2 = 0.9996	3.2×10^{-2}-4	14.50
**25**	605.2189$[M+Na]^+$	y = 783548x - 5838.1	R^2 = 0.9999	2.6×10^{-2}-1.3	3.71
**26**	603.2039$[M+Na]^+$	y = 4×10^6x - 4614.9	R^2 = 0.9992	1.8×10^{-3}-0.18	6.78

Table S4. Results of liner relationship of additive contents

Additive	λ_{max} (nm)	M/Z	Line equation	R^2	Linear range
benzoic acid	228	145.026[M+Na]$^+$	y = 60695x + 64306	0.9993	2.5-40
ethylparaben	255	189.052[M+Na]$^+$	y = 51804x + 5634.1	0.9999	0.625-10
sorbic acid	259	135.041[M+Na]$^+$	y = 130523x - 2052	0.9996	1.25-20

Table S5. Results of liner relationship of carbohydrate

Carbohydrate	Linear equation	R^2	Linear range
arabinose	y = 64.25x + 32.686	0.9991	4.34-63.40
glucose	y = 62.806x - 89.899	0.9994	2.26-132.60
xylose	y = 72.615x - 55.297	0.9991	2.40-74.10
mannose	y = 50.744x - 8.7535	0.9996	1.14-33.40

Compound **1**

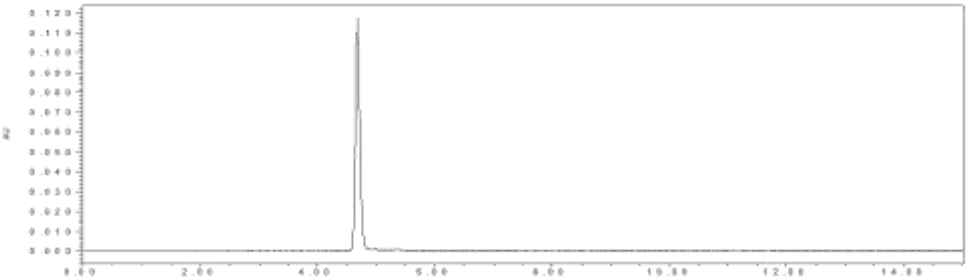

Compound **2**

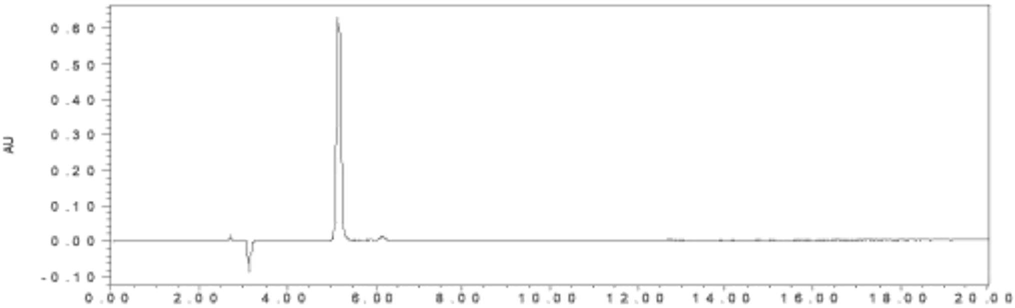

Compound **3**

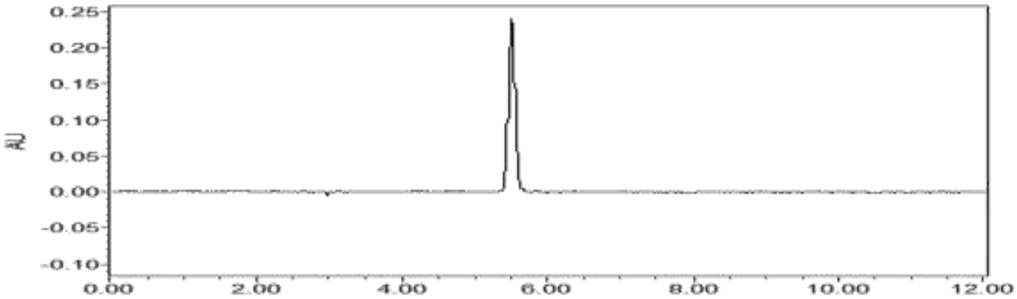

Compound **4**

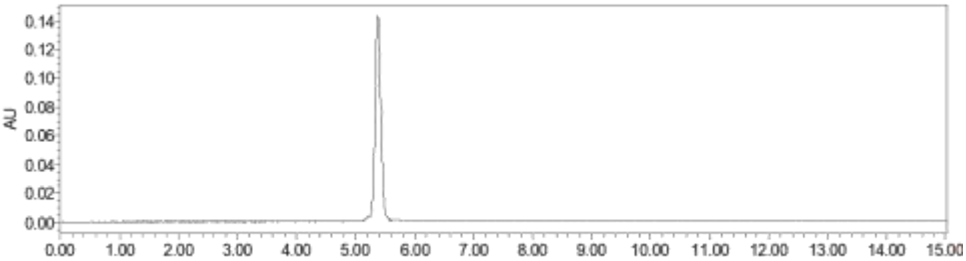

Compound **5**

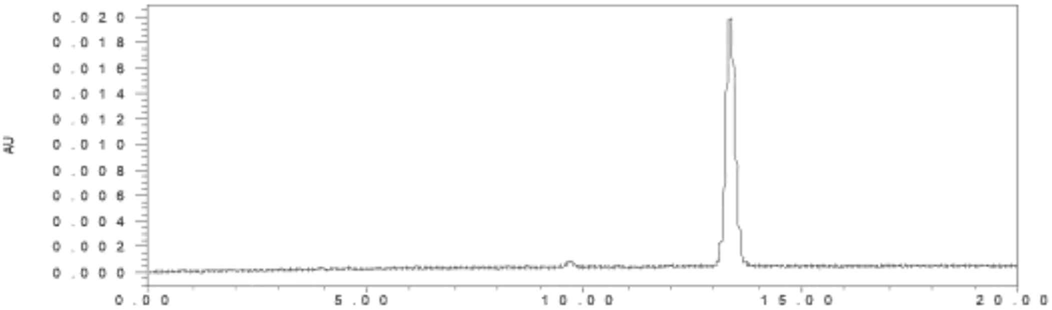

Compound **6**

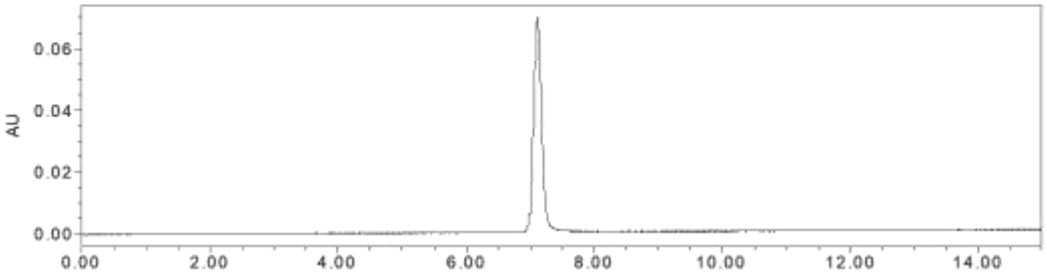

Compound **7**

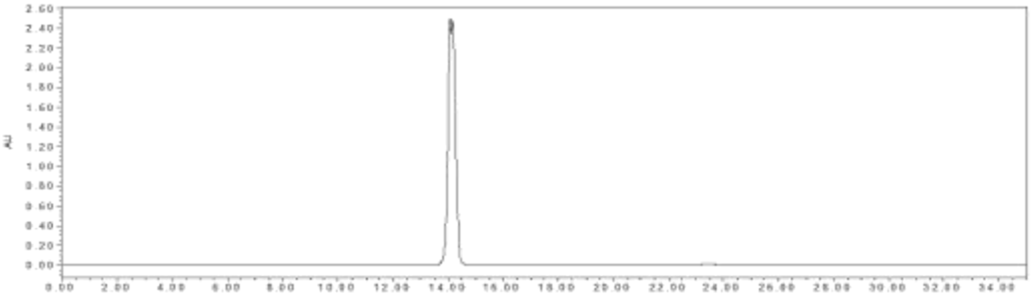

Compound **8**

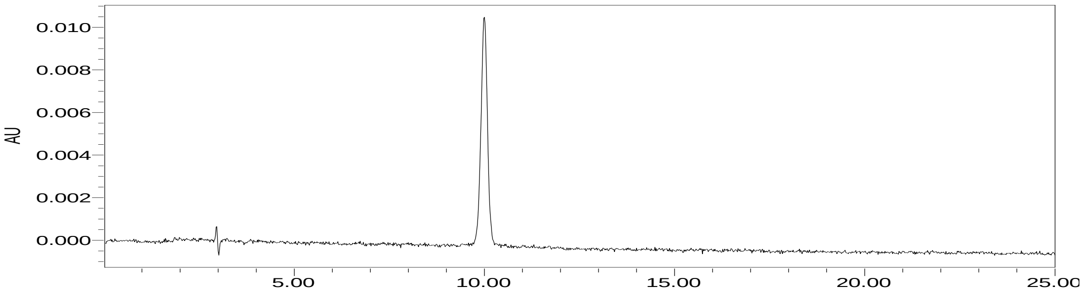

Compound **9**

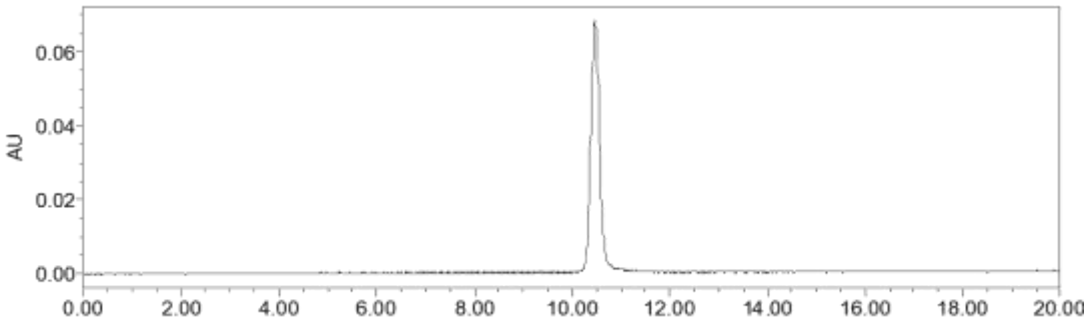

Compound **10**

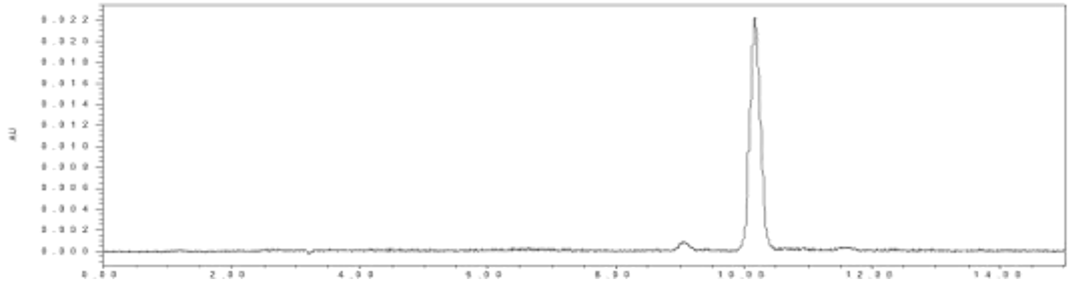

Compound **11**

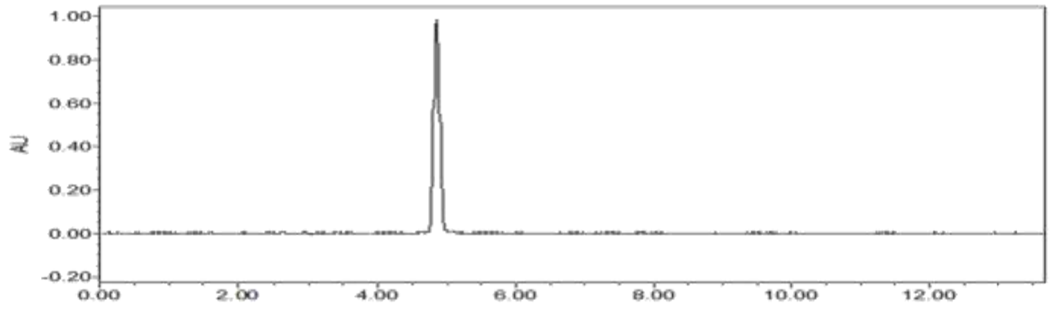

Compound **12**

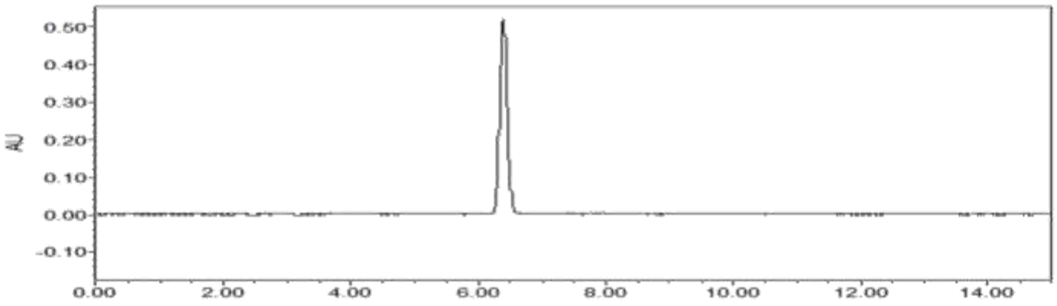

Compound **13**

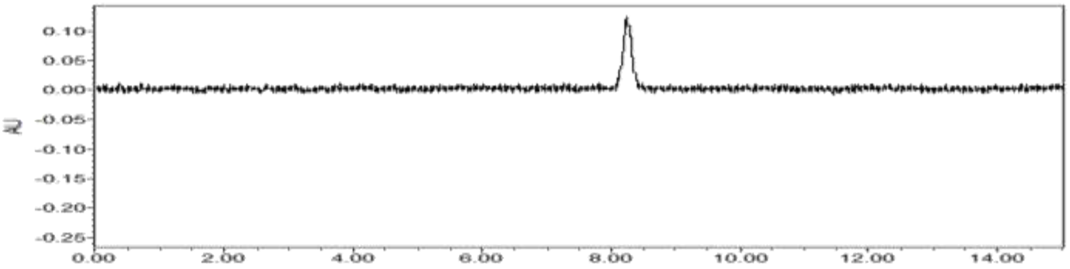

Compound **14**

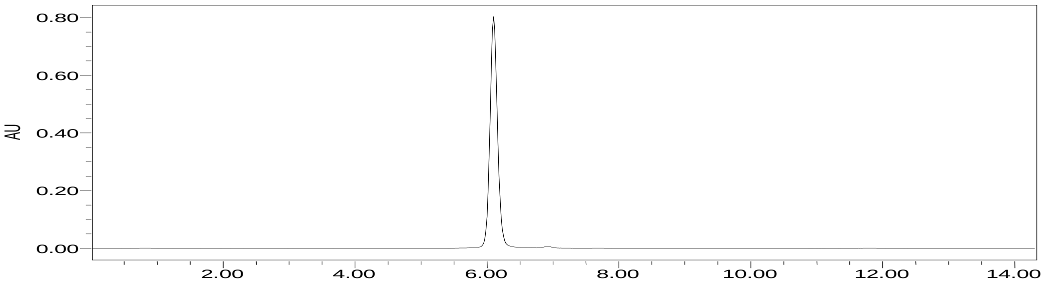

Compound **15**

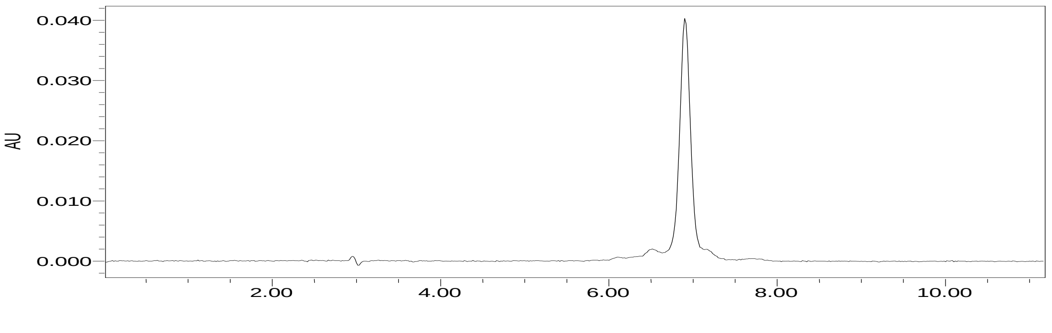

Compound **16**

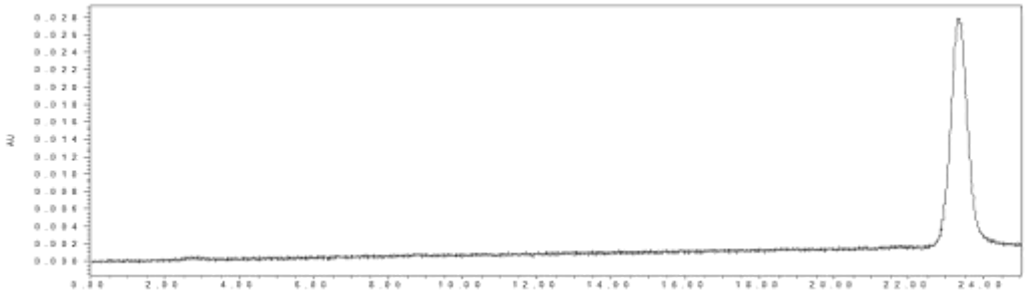

Compound **17**

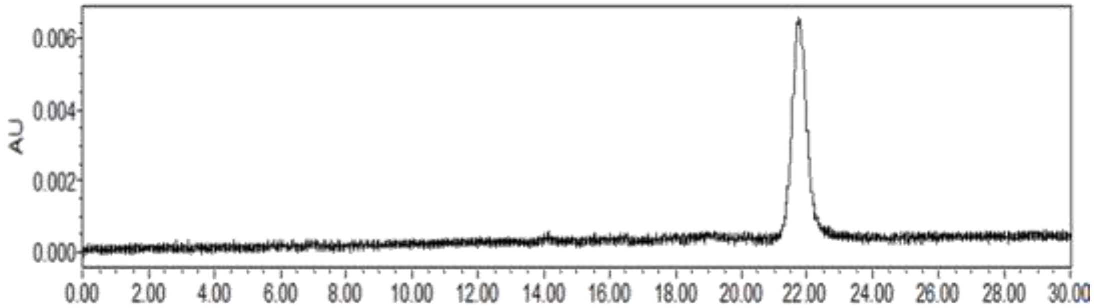

Compound **18**

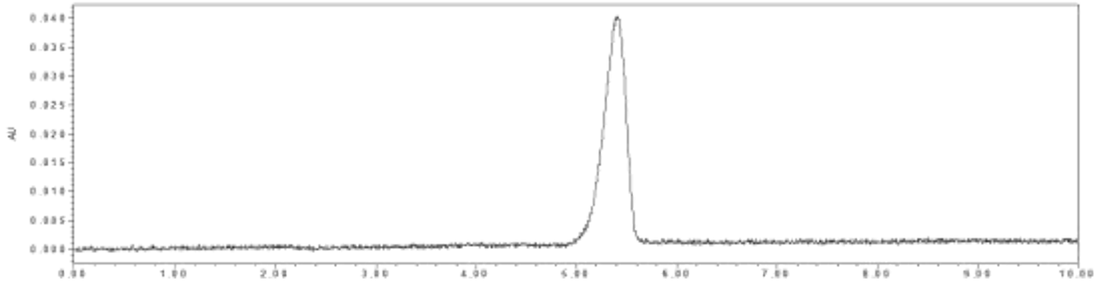

Compound **19**

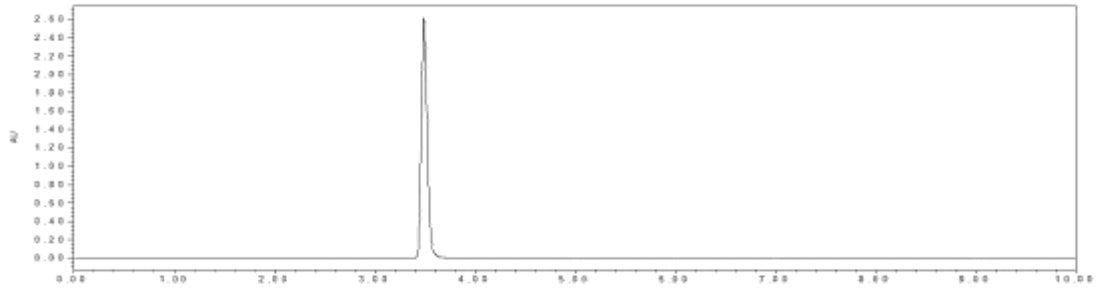

Compound **20**

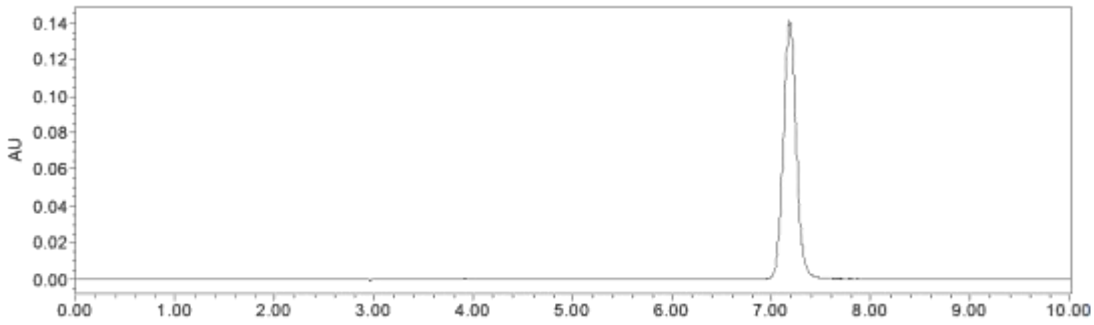

Compound **21**

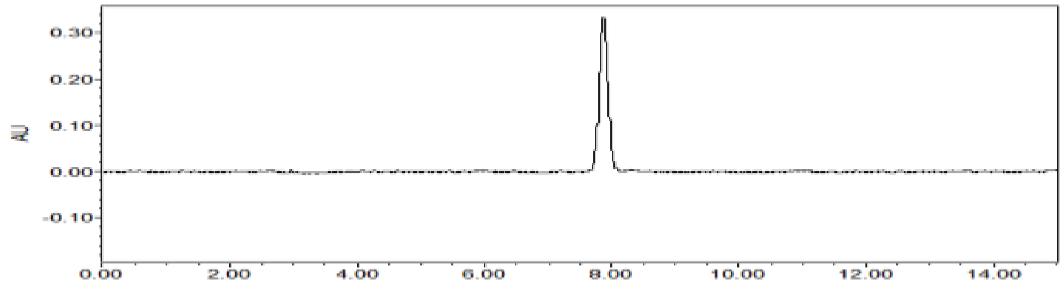

Compound **22**

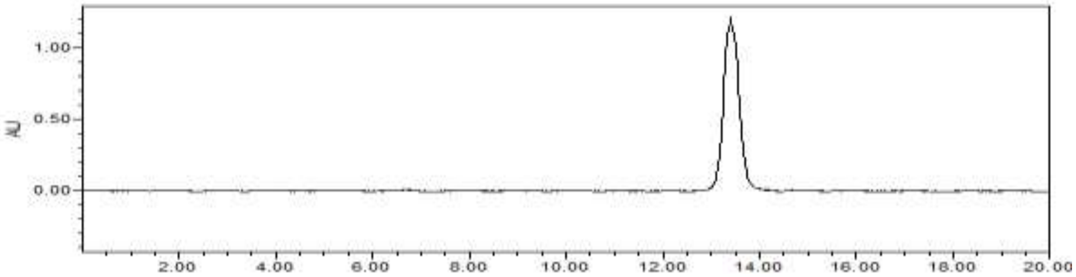

Compound **23**

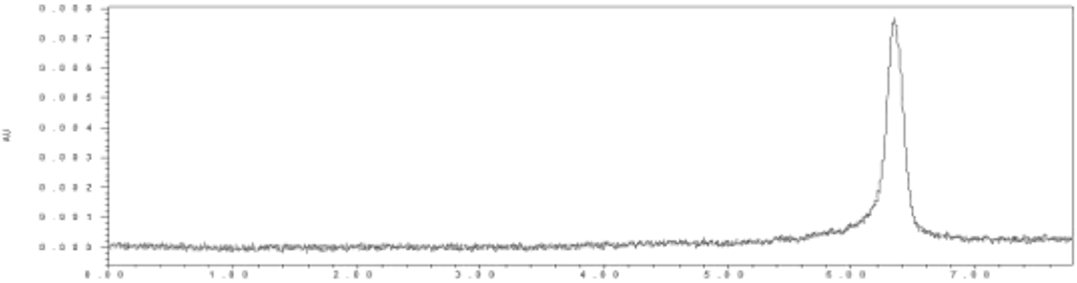

Compound **24**

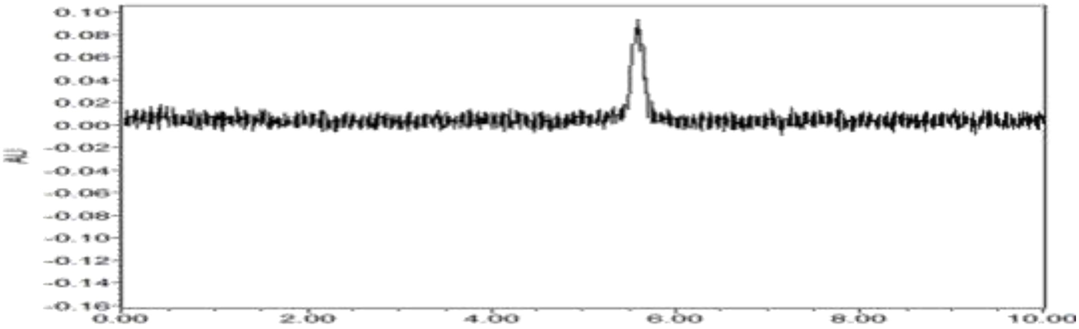

Compound **25**

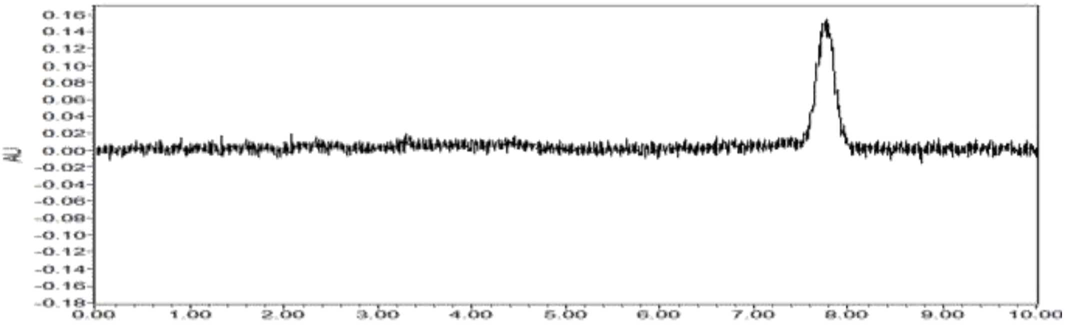

Compound **26**

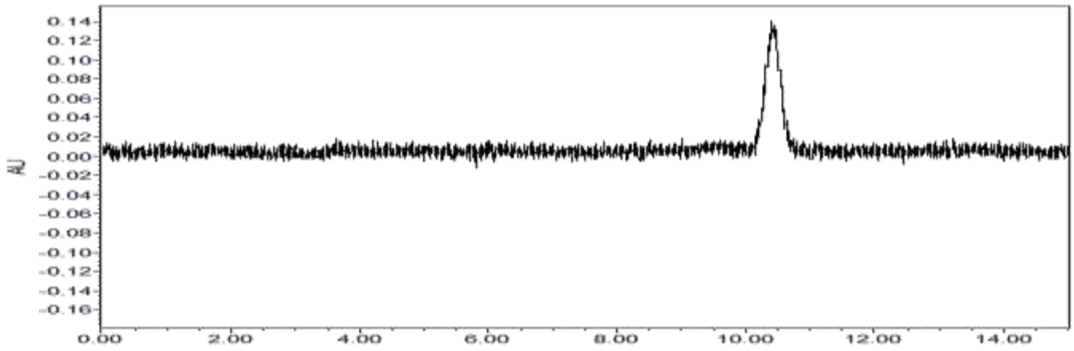

Figure S1. Compounds **1-26** purification verification chromatography

Additive a

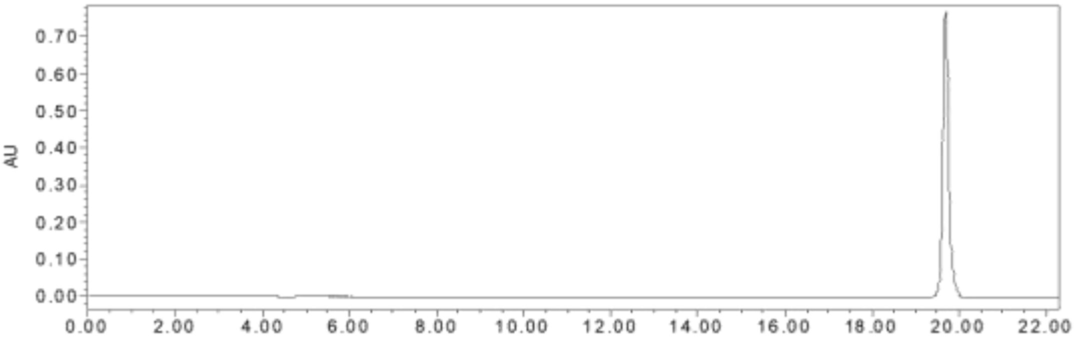

Additive b

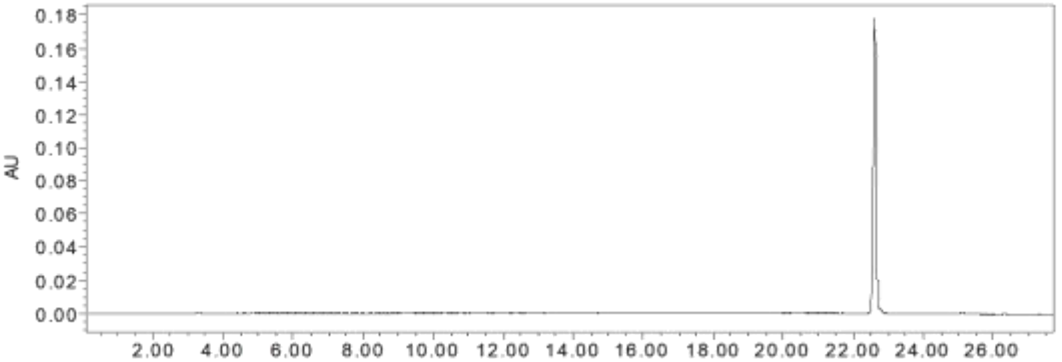

Additive c

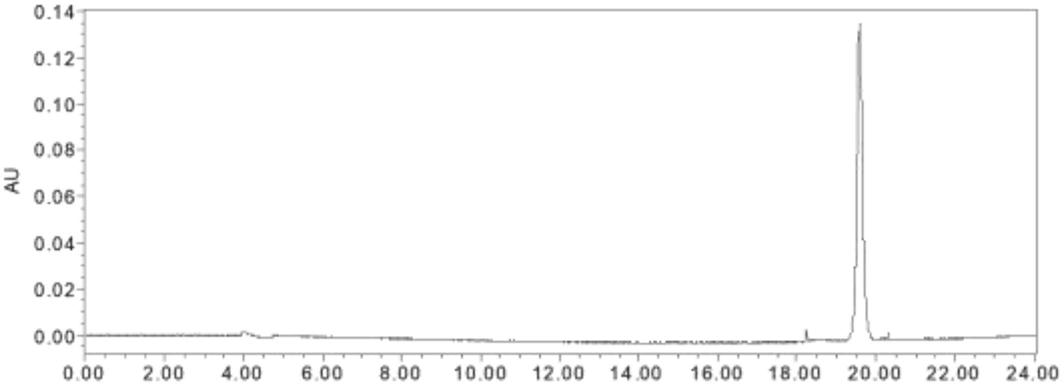

Figure S2. HPLC chromatography of three preservatives

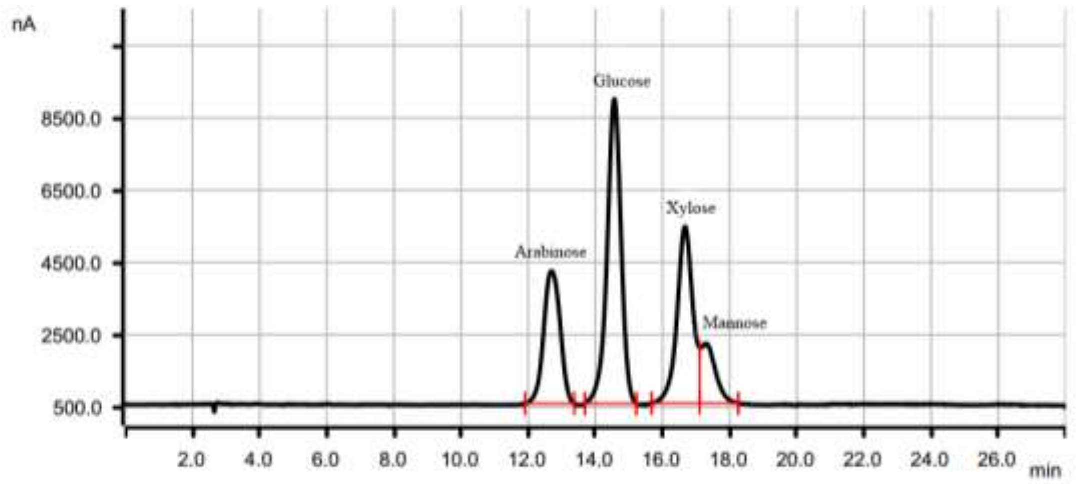

Figure S3. Ion chromatogram of carbohydrate
